# Cathelicidin Host Defense Peptides and Inflammatory Signaling: Striking a Balance

**DOI:** 10.3389/fmicb.2020.01902

**Published:** 2020-08-27

**Authors:** Morgan A. Alford, Beverlie Baquir, Felix L. Santana, Evan F. Haney, Robert E. W. Hancock

**Affiliations:** ^1^Centre for Microbial Diseases and Immunity Research, University of British Columbia, Vancouver, BC, Canada; ^2^Departamento de Medicina Molecular y Bioprocesos, Instituto de Biotecnología, Universidad Nacional Autónoma de México, Cuernavaca, Mexico

**Keywords:** host-defense peptide, innate immunity, homeostasis, toll-like receptor, self-antigen

## Abstract

Host-defense peptides (HDPs) are vital components of innate immunity in all vertebrates. While their antibacterial activity toward bacterial cells was the original focus for research, their ability to modulate immune and inflammatory processes has emerged as one of their major functions in the host and as a promising approach from which to develop novel therapeutics targeting inflammation and innate immunity. In this review, with particular emphasis on the cathelicidin family of peptides, the roles of natural HDPs are examined in managing immune activation, cellular recruitment, cytokine responses, and inflammation in response to infection, as well as their contribution(s) to various inflammatory disorders and autoimmune diseases. Furthermore, we discuss current efforts to develop synthetic HDPs as therapeutics aimed at restoring balance to immune responses that are dysregulated and contribute to disease pathologies.

## Introduction

Host defense peptides (HDPs) have evolved across all species of animals and are recognized as vital components of innate immune processes (Haney et al., 2019a; Mookherjee et al., 2020). HDPs are short, gene-encoded polypeptides (10–50 residues in length) that are broadly characterized by a net positive charge and a high proportion of hydrophobic amino acids (Fjell et al., 2012). They can exhibit potent bactericidal activity in buffer, which is why they are often referred to as antimicrobial peptides (AMPs), although this activity is often abrogated by host physiological conditions including (especially divalent) cation concentrations and the presence of polyanions such as glycosaminoglycans (Bowdish et al., 2005a; Hancock et al., 2016). Conversely, under host-like physiological conditions and in animal models, many natural AMPs are able to modulate the host innate immune response. Indeed, the immunomodulatory activity of these molecules might be more representative of their natural functions and potential for development as therapeutic agents. Numerous studies have focused on unraveling the mechanisms that underlie the various immunomodulatory functions of HDPs in diverse scenarios (Davidson et al., 2004; Chen Y. et al., 2017; Chen S. et al., 2018). While no general mechanism has been described for all HDPs, several features of the immunomodulatory response to HDPs have been described for a variety of cell types and animal models, including cellular recruitment, anti-inflammatory activity, and wound healing, among others (Hancock et al., 2016).

Current knowledge about the activities of HDPs has been largely derived from the study of naturally-occurring peptides from vertebrates (Van Dijk et al., 2018). Some HDPs are expressed constitutively by immune cells, whereas the local concentration of others can be upregulated in response to a particular stimuli and/or secreted into the local environment or released from phagocytes by degranulation (Elloumi and Holland, 2008). Several HDPs are also expressed by epithelial, cells of the skin, gastrointestinal, genital, and respiratory tracts as well as a variety of other cell types (Lee et al., 2016). The most abundant and best characterized HDPs in mammals are those classified as cathelicidins and defensins (Fruitwala et al., 2019). Numerous cathelicidins have been described in mammals as well as other phyla including birds, reptiles, amphibians, and fish (Uzzell et al., 2003; Xiao et al., 2006; Van Harten et al., 2018).

Here we focus on the features of cathelicidins that contribute to their immunomodulatory properties and highlight the potential for developing synthetic HDP derivatives as novel therapies for various inflammatory conditions. An overview of the structure, function, and expression of naturally-occurring cathelicidins across vertebrates is provided with a particular emphasis on their ability to maintain homeostasis by influencing immune signaling and mitigating damaging inflammatory responses (Mookherjee et al., 2006). In addition, we discuss disorders that are made more severe by cathelicidins acting as self-antigens, and describe various diseases associated with dysregulated expression of cathelicidins. Several examples of synthetic peptides that have been designed to harness the beneficial effects of natural peptides are highlighted, particularly for their capacity to modulate innate immune processes (Hilchie et al., 2013). In addition, we examine an emerging role for cathelicidins and synthetic HDP derivatives in the management of dysregulated immunity present in sepsis (Martin et al., 2015). Finally, we highlight several ongoing clinical trials aimed at exploiting the immunomodulatory functions of HDPs and discuss emerging peptide formulation strategies and studies in animal models that bridge the gap between pre-clinical and clinical development of novel peptide therapies.

## Evolutionary Perspectives of Cathelicidins Across Vertebrate Species

**FIGURE 1:**
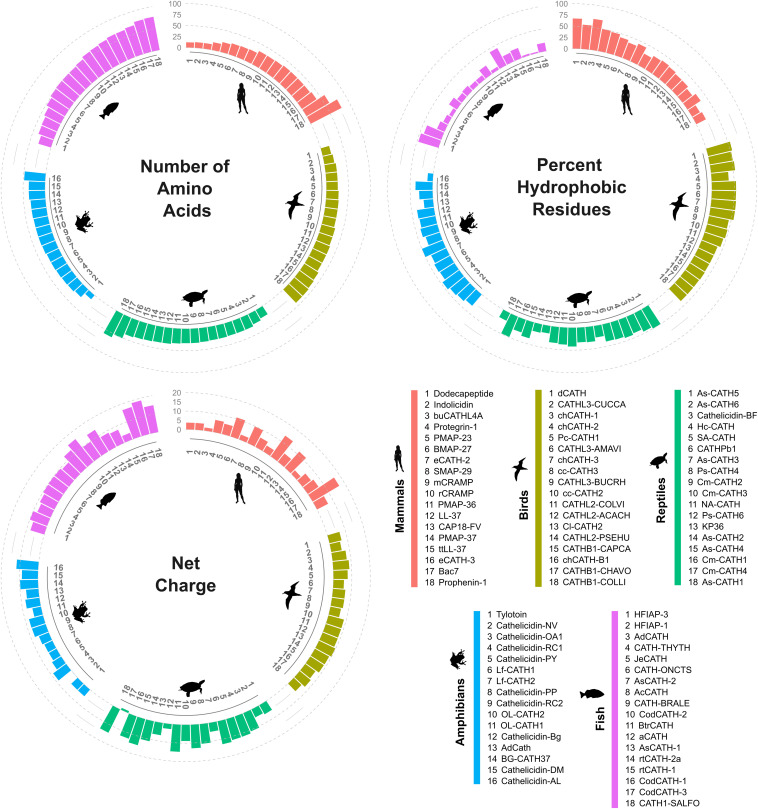
Diversity of cathelicidin peptides among vertebrate groups. Circular bar plots show the distribution of length, charge and proportion of hydrophobic amino acids among representative vertebrate cathelicidin peptides (see Supplementary Table S1). The order of sequences in each group is sorted by peptide length. Physico-chemical properties were computed using the *Peptides* package v2.4.2 (Osorio et al., 2015) in R v4.0.0 (R Core Team, 2020). Net charge was predicted using the Bjellqvist’s pK scale implemented in the *Peptides* package. Animal silhouettes were created by: NASA (mammals; human), Juan Carlos Jerí (birds; shearwater), uncredited (reptiles; turtle), Will Booker (amphibians; tree frog) and Felix Vaux (fish), and downloaded from http://phylopic.org/.

The cathelicidin family of HDPs exhibits a broad diversity in structure and function across all vertebrates. The number of genes encoding cathelicidin analogs can vary by species. For instance, only a single cathelicidin gene is encoded in humans, mice, and dogs, while 2–11 cathelicidin-coding genes have been identified in certain species of fish, amphibians, reptiles, birds, and most other mammals (Ramanathan et al., 2002; Masso-Silva and Diamond, 2014; Kim et al., 2017; Qi et al., 2019). The organization of the coding sequence seems to be well conserved among vertebrates and is comprised of four exons that collectively encode the precursor peptide consisting of a signal peptide sequence, the cathelin pro-domain, and the mature cathelicidin sequence (Ramanathan et al., 2002; Dalla Valle et al., 2013). Although there is high amino acid sequence identity for the cathelin domain between species, the mature form of the cathelicidin peptide is remarkably diverse in length, composition, net charge, and structure (Figure 1).

Mature cathelicidin peptides can be loosely grouped into four structural classes: α-helical or linear peptides that can adopt helical conformations under physiological conditions or in the presence of biological membranes; linear peptides that are disproportionately high in particular amino acids such as glycine, serine, proline or tryptophan; and two classes stabilized by disulfide bridges, namely β-structured and cyclic peptides (Zanetti, 2005). The α-helical peptides are the most widely distributed and present in all vertebrate groups, but other structural classes are observed across species (Masso-Silva and Diamond, 2014; Chen Y. et al., 2017 see Supplementary Material).

It has been suggested that HDPs found in multicellular organisms arose as a protective mechanism against microbes, particularly against bacteria (Boman, 2003; Lazzaro et al., 2020). In such a scenario, it is assumed that host-microbial interactions and direct antimicrobial activity drove the evolution of HDP sequences to optimize them collectively for anti-bacterial potency. However, as mentioned above, the antimicrobial potency of most HDPs remains rather modest in host-like environments. A recent study of mammalian homologs of LL-37 proposed that the driving force behind the evolution of cathelicidins might be their interaction with host receptors (Zhu and Gao, 2017), which is consistent with the concept that immune response elements are one of the most highly evolving groups of proteins across mammalian species (Hahn et al., 2007; Kosiol et al., 2008).

An earlier study suggested that the disordered C-terminus of LL-37 interacts with the N-formyl peptide receptor-like (FPR) family of proteins (Singh et al., 2014), as part of the process to mediate chemotaxis. Sequence analysis of the human FPR2 receptor indicated high variability in the ligand-binding extracellular loop domain, while the C-terminus of mammalian LL-37 homologs was disordered; thus statistical analysis revealed a possible co-evolution of this peptide as a cognate binding partner for FPR2 was proposed (Zhu and Gao, 2017). Furthermore, the elimination of the disordered N- and C-terminal regions in the rabbit LL-37-homolog (CAP18-FV) or their replacement with disordered regions from the dolphin (ttLL-37) or human homologs had no impact on the anti-bacterial activity. Unfortunately, since the immunomodulatory properties of the resulting species-hybrid mutants of LL-37 were not evaluated, the direct influence of this proposed interaction was not confirmed. Regardless, several other host receptors with immune functions have been proposed to interact with cathelicidins, including purinergic receptors P2Y11 and P2 × 7, the CXC chemokine receptor type 2, Mas-related gene X2 (MrgX2), GAPDH, and others (Verjans et al., 2016). This provides strong evidence that receptor binding directly impacts the biological functions of cathelicidins. Curiously, a similar evolutionary analysis did not identify highly variable residues in avian cathelicidins, suggesting that this putative co-evolutionary relationship might be specific to mammalian LL-37 homologs (Cheng et al., 2015).

## Regulation of Cathelicidin Expression

Since the repertoire and cell/tissue distribution of cathelicidins varies by species, we focus below on discussing the expression and activity of the human cathelicidin antimicrobial peptide (*CAMP*) gene found on chromosome 3p21 (Elloumi and Holland, 2008). The *CAMP* gene encodes the 18 kDa precursor human cationic antimicrobial protein, hCAP18, which is cleaved by proteases to generate the active peptide known as LL-37. It is expressed in a variety of tissues and cell types, including epithelial cells and many cells of the immune system (Hancock et al., 2016). Expression of hCAP18 is highest in the bone marrow in healthy individuals (Fagerberg et al., 2014), although expression can be detected in many organs and tissues. Secretory glands enhance basal expression at mucosal surfaces, with hCAP18 secreted in the semen, saliva, and sweat (Andersson et al., 2002). Most studies of the regulation of *CAMP* expression in various tissues reflect recognition of inflammatory stimuli by neutrophils and monocytes, since these cell types produce more hCAP18/LL-37 than other immune cells. In addition, neutrophils store the inactive hCAP18 precursor in specific (azurophilic) granules for rapid deployment during immune responses (Kai-Larsen and Agerberth, 2008). Recognition of inflammatory signals leads to cascading activation of immune cells and an increase in *CAMP* expression, particularly in leukocytes, as well as LL-37 secretion due to neutrophil degranulation. Increased expression of *CAMP* has been attributed to endoplasmic reticulum (ER) stress which is in part associated with NF-κB activation and concomitant downstream signaling events (Park et al., 2011), although other factors aside from ER stress might contribute to enhanced *CAMP* expression following inflammatory stimulus. Consistent with this, LL-37 production is induced by a variety of inflammatory disorders that are not associated with infection (Kahlenberg and Kaplan, 2013), and exogenous host defense metabolites, such as short chain fatty acids and butyrate, which strongly induce *CAMP*/LL37 expression (Chen and Vitetta, 2020). During secretion, proteinase 3 or kallikreins, produced by monocytes or cells at the skin surface, respectively, cleave the precursor hCAP18 protein to generate the active LL-37 peptide as well as truncated forms with varying biological activities (Murakami et al., 2004; Yamasaki et al., 2006).

Beyond the enhanced production of hCAP18 in response to inflammation and pathogen exposure, a growing body of research is addressing *CAMP* expression following exposure to physiologically-important metabolites (Coorens et al., 2017). The *CAMP* promoter is directly targeted by the cognate vitamin D receptor (VDR) found in various tissues, and thus vitamin D_3_ and its metabolites can induce widespread *CAMP* expression, especially in myeloid cells (Wang et al., 2004). For example, the hormonal form of vitamin D_3_, 1,25-dihydroxyvitamin D_3_, upregulates the expression of *CAMP* in immortalized human keratinocytes, acute myeloid leukemia, and colon cancer cell lines as well as in primary bone marrow derived macrophages (Gombart et al., 2005). Combining exogenous vitamin D_3_ with cytokines, such as IL-13, that favor T_*h*_2 polarization of CD4^+^ T cells, further enhances this VDR-mediated *CAMP* expression (Schrumpf et al., 2012).

## Biological Role of Cathelicidins

### LL-37 and mCRAMP

**FIGURE 2:**
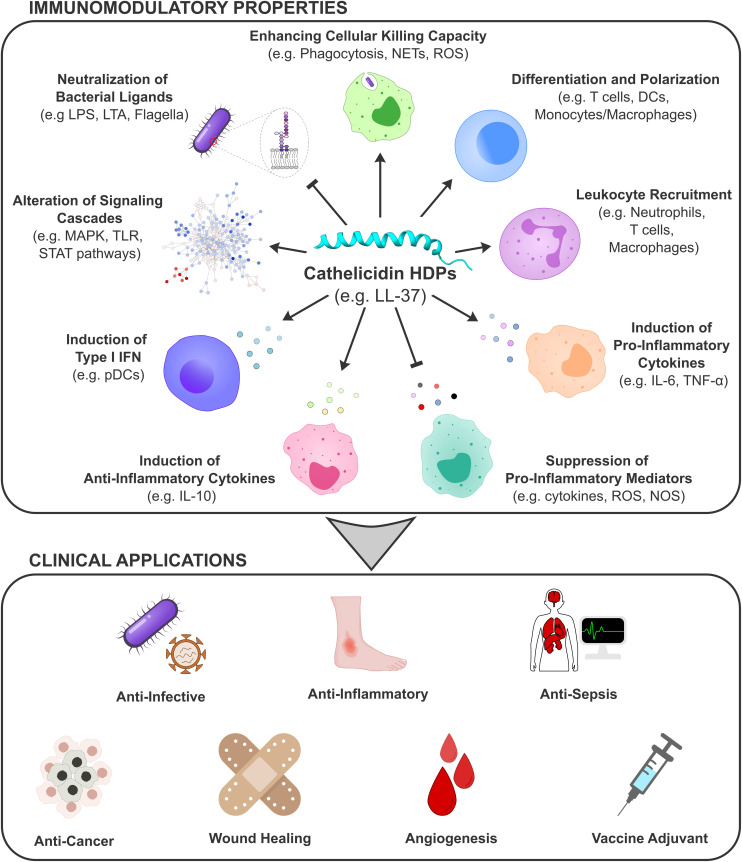
The immunomodulatory features of cathelicidins. Immunomodulatory functions of cathelicidin HDPs include, but are not limited to: enhancing cellular killing capacity, differentiation and polarization of immune cells, leukocyte recruitment, induction or suppression of pro-inflammatory mediators, induction of anti-inflammatory cytokines, induction of type I IFN, alteration of signaling cascades and neutralization of bacterial ligands. These functions can be harnessed for anti-infective, anti-inflammatory, anti-sepsis and anti-cancer applications or contribute to wound healing, angiogenesis and improved vaccine effectiveness. DCs, dendritic cells; IFN, interferon; IL, interleukin; LPS, lipopolysaccharide; LTA, lipotechoic acid; MAPKs, mitogen-activated protein kinases; NOS, nitrous oxide species; ROS, reactive oxygen species; STAT, signal transducer and activator of transcription; TLRs, toll-like receptors; TNF, tumor necrosis factor.

HDPs, in general, exert an incredible array of immunomodulatory functions and many of these features are shared by members of the cathelicidin family of peptides (Figure 2), although individual peptides tend to favor a subset of these properties (reviewed in ref 4). Of the many biological functions of HDPs, their antimicrobial functions have undoubtedly been the most widely researched in part due to the simple assays involved. While many studies have emphasized the important role of cathelicidins as antimicrobials at epithelial surfaces, particularly the skin (Travis et al., 2000), such conclusions must be qualified due to the conditions under which such activities were assessed, often in very dilute salts. For instance, phosphate buffer, in which many of these studies were undertaken, is decidedly not physiological since *in vivo* conditions involve high concentrations of divalent and monovalent cations and polyanionic sugars that can inhibit antimicrobial activity (Bowdish et al., 2005b).

Admittedly, studies in mice deficient in the cathelin-related antimicrobial peptide (mCRAMP), the murine ortholog of LL-37, have demonstrated an enhanced susceptibility to a variety of infections including necrotizing skin infections caused by Group A *Streptococcus* (Nizet et al., 2001), keratitis produced by *P. aeruginosa* (Huang et al., 2007), and meningitis induced by *Streptococcus pneumoniae* (Merres et al., 2014). However, it is possible that such studies could reflect the immunomodulatory effects of cathelicidins that enables protection against infections (Bowdish et al., 2005b; Hancock et al., 2016). In the following section, we discuss studies demonstrating that cathelicidins, such as LL-37, have a primary role in modulating the (innate) immune response which is robust, complex, and occurs under physiological conditions both *in vitro* and in animal models.

One outstanding feature of LL-37 is its ability to suppress pro-inflammatory signaling. This likely involves a complex series of both direct and indirect mechanisms (Koo and Seo, 2019). Regarding direct mechanisms, cathelicidins bind to and neutralize the bacterial toll-like receptor (TLR) ligands, such as lipopolysaccharide (LPS) or lipoteichoic acid (LTA) (Kandler et al., 2006), which would otherwise engage TLRs and trigger inflammatory processes associated with cascading activation of immune cells (Horibe et al., 2013). In addition, LL-37 can substantially attenuate LPS-mediated TNF-α production from peripheral blood mononuclear cells (PBMCs) when added either before or after the LPS stimulus, consistent with the notion of a variety of indirect mechanisms that reflect LL-37 uptake into cells and modulation of various intracellular signaling events (Hilchie et al., 2013). Indeed, in PBMCs challenged with TLR-2, TLR-4, or TLR-9 agonists, LL-37 generally suppresses the production of the pro-inflammatory cytokines TNF-α and IL-1β and alters expression of IL-6 and IL-8 (Mookherjee et al., 2006; Hilchie et al., 2013). Moreover, based on microarray analysis, more than 160 genes up-regulated in LPS-stimulated THP-1 monocytic cells were suppressed in LL-37 treated cells (Mookherjee et al., 2006). Thus, LL-37 neutralizes bacterial signature molecules (TLR agonists) that normally induce pro-inflammatory immune responses.

Several lines of evidence support the host-cell-directed activity of cathelicidins that is independent of bacterial ligand binding. For example, LL-37 modulates more than a dozen signaling pathways including the p38, Erk1/2, JNK MAP-kinases, NFκB, PI3K/Akt, Src family kinase, TRIF-IRF, TREM, Wnt/β-Catenin, JAK-STAT, and autophagy signaling pathways independent of LPS, LTA and/or flagellin stimulation (Scott et al., 2002; Mookherjee et al., 2009; Hancock et al., 2016). Furthermore, systems biology and biochemical studies on human CD14^+^ monocytes treated with LL-37 showed nearly 500 genes changing expression, reflecting the involvement of many of these pathways in chemokine induction in response to LL-37 (Mookherjee et al., 2009). Activation of Wnt/β-Catenin and PI3K/Akt signaling cascades elicited by LL-37 was also demonstrated in samples from non-small cell lung carcinoma patients (Ji et al., 2019) with a positive correlation between LL-37 concentration in tissues and relevant gene expression. Similarly, in the A549 pneumocyte cell line *in vitro*, LL-37 stimulated growth through a β-Catenin-dependent but TLR-independent manner (Ji et al., 2019). Overall, such studies provide evidence that LL-37 has multiple surface and intra-cellular targets (Hilchie et al., 2013; Levast et al., 2019) that contribute to the biological functions of this HDP *in vivo*, resulting in diverse outcomes mediated by a broad array of signaling events (Figure 2).

A typical outcome is the induction of certain chemokines in a variety of cell types, including phagocytes and epithelial cells. Although the resting concentrations of LL-37 at mucosal surfaces can be quite low, local LL-37 concentrations at sites of infection or during acute inflammation can be much higher due to degranulation of phagocytes releasing LL-37 into the vicinity. The high local concentration promotes the well-studied ability of cathelicidins to enhance the recruitment of immune cells, especially phagocytes (monocytes, macrophages, and neutrophils), to the sites of infections in mice (Hilchie et al., 2013). For example, the release of LL-37 by human keratinocytes activate the Src family kinases and enhance TLR-5 activation upon flagellin stimulation, thereby inducing the release of chemokines (Nijnik et al., 2012). LL-37 and other HDPs can also act as chemokines to directly attract immune cells. Thus, binding of the FPR2 receptor by LL-37 enables the recruitment of neutrophils, monocytes, T cells, and mast cells (Yang et al., 2000) although, generally speaking, this occurs at higher peptide concentrations than other chemokine inducing activities, likely due to low ligand binding affinity.

However, not all of the functions of LL-37 are beneficial. For example, LL-37 promotes histamine release from mast cells, which promotes loosening of blood vessel walls to enable enhanced uptake of immune cells, but this process is also allergenic (Niyonsaba et al., 2002). Mast cell degranulation (and histamine release) is mediated by LL-37-induced activation of the MrgX2 receptor that triggers a range of signaling pathways such as PI3K/Akt, Erk1/2, and JNK (Yu et al., 2017). LL-37 also promotes microbially-induced apoptosis of epithelial cells while extending the lifetime of neutrophils (Barlow et al., 2010), which could contribute to damage associated with respiratory infections.

TLRs play a crucial role in innate immunity and govern pathogen recognition by and activation of important sentinel cells, such as macrophages, neutrophils, dendritic cells (DCs), and epithelial cells (Beutler, 2009). In addition to their ability to suppress proinflammatory signaling through TLRs, cathelicidins can also influence the expression of TLRs in a variety of cell types including mast cells, monocytes, neutrophils, renal cells, epithelia lining the colon, and other mucosal surfaces (Agier et al., 2018). Interestingly, the influence of LL-37 on TLR expression is both tissue- and time- dependent. For example, protein levels of TLR-2, TLR-4, TLR-5, and TLR-9 increased in Wistar rat mast cells in the presence of LL-37 in a time-dependent manner and peaked 3 hours following stimulation *in vitro* (Agier et al., 2018). Furthermore, LL-37 downregulated TLR and co-receptor expression induced by LPS in human gingival fibroblasts, but did not influence expression in untreated cells (Inomata et al., 2019), although the relevance of this is unknown since, as mentioned above, LL-37 strongly reduces LPS/LTA-mediated proinflammatory cytokine expression (Kandler et al., 2006).

Cathelicidins can alter the host immune response prior to, during, and even after infection. By directly influencing lymphocytes while also altering the chemokine profile associated with T and B cells, as well as affecting innate immune responses (e.g., cytokines, etc.) that prime and activate lymphocytes, cathelicidins link the innate and adaptive immune systems (Mookherjee et al., 2009; Nicholls et al., 2010). LL-37 and its derivatives also have the ability to induce differentiation and maturation of DCs as well as activate plasmacytoid DCs (pDC) and macrophages that prime and activate adaptive immunity (Davidson et al., 2004; Kandler et al., 2006). Overall, the features of LL-37 that promote modulation of the immune system, rather than direct microbicidal effects, seem to assist the resolution of an infection while also regulating harmful inflammation. The immunomodulatory effects of cathelicidins include selectively enhancing and diminishing inflammation by direct and/or indirect chemotaxis, pro-inflammatory and anti-inflammatory cytokine production, blocking TLR activation and downstream signaling pathways, and promoting activation of adaptive immunity.

### Cathelicidins From Other Vertebrates

Although human LL-37, and to a lesser extent mCRAMP, have been the most highly studied HDPs (Van Harten et al., 2018), cathelicidins are ubiquitous in vertebrates. To determine similarities between the biological activities of natural cathelicidins, Coorens et al. (2017) compared the activity of 12 cathelicidins from chickens and five different mammalian species (human, mouse, dog, horse, and pig). They found that some functions of cathelicidins, such as suppression of bacterial ligand-induced TNF-α secretion and modest antimicrobial activity, were largely conserved. Therefore, while they speculated that these might represent the “core” biological activities of cathelicidins across vertebrate species, the above-mentioned caveat about limited antimicrobial activity under physiological conditions is worth taking into account. Critically, studies comparing the various biological activities of cathelicidins from different vertebrate groups under the same experimental conditions are lacking. We contend that the massive sequence and structural variability of cathelicidins (and more generally HDPs), is the result of a complex evolutionary process where different peptides have evolved toward similar functions. It is expected that the individual host-specific activities of HDPs will differ, especially since each species produces its own repertoire of HDPs for which expression varies depending on tissue distribution and immune stimulus. Thus, the overall function of HDPs would be the important evolutionary driver and one might not expect any given peptide to have a single optimized purpose.

The immunomodulatory activities of non-human and non-murine cathelicidins are usually tested against human- or mouse-derived cell lines, which may obscure host-specific responses and functions of the peptides. However, functional insights into the biological roles played by these cathelicidins in their native hosts can still be gleaned by these types of studies. In the following section, we highlight some of the research related to the lesser studied cathelicidin peptides from other vertebrate groups and highlight several conserved activities that contribute to their likely biological function and therapeutic potential.

In amphibians, the skin plays a major role in water regulation, respiration, and defense. However, it also constitutes a permissive environment for potential pathogens, being rich in water, nutrients, and oxygen to enable growth (Clarke, 1997). To protect themselves, amphibians secrete a wide assortment of bioactive peptides, including HDPs, with diverse functions (Xu and Lai, 2015). In this regard, several amphibian cathelicidin peptides have been identified, and many of these have potent immunomodulatory functions (Mu et al., 2014; Cao et al., 2018; Wu et al., 2018; Shi et al., 2020). For example, cathelicidin-PP is an inducible β-structured cathelicidin from the skin secretions of the tree frog *Polypedates puerensis* that can significantly inhibit the production of nitric oxide (NO) and pro-inflammatory cytokines [TNF-α, interleukins (IL)-1β and IL-6] by LPS-stimulated mouse peritoneal macrophages (Mu et al., 2017). The suppression of pro-inflammatory mediators was proposed to be mediated through direct binding of LPS, but also led to decreased phosphorylation of MAP kinases and inhibition of NF-κB signaling pathways (Mu et al., 2017).

Several cathelicidin peptides have been found in reptiles, often exerting broad antimicrobial activities toward bacteria and fungi (Dalla Valle et al., 2013; Chen Y. et al., 2017; De Barros et al., 2019; Qiao et al., 2019). Recent studies have also demonstrated that they inhibit biofilm formation and can eradicate pre-formed biofilms of various species of bacteria (Chen Y. et al., 2017; De Barros et al., 2019; Qiao et al., 2019). In addition, reptilian cathelicidins typically possess low hemolytic and cytotoxic activity against mammalian cells and can inhibit production of LPS-induced NO and pro-inflammatory cytokines by mammalian macrophages *in vitro*. These inhibitory activities are usually ascribed to direct binding of the peptide to LPS and to the TLR-4/MD2 complex, which prevents receptor activation and inhibits MAP kinase and NF-κB signaling (Chen Y. et al., 2017; Qiao et al., 2019), although direct inhibition of signaling responses is frequently not examined. On the other hand, the induction of chemokines like MCP-1 and anti-inflammatory cytokines like IL-10 has been observed for several cathelicidins across reptilian species (Cai et al., 2018; Shi et al., 2019).

Reptilian cathelicidins can also influence immune processes without directly modulating cytokine signaling. For example, the snake cathelicidin-WA/-BF (CWA) promoted M2-like polarization of *E. coli-*stimulated murine macrophage RAW264.7 cells (Chen S. et al., 2018). When co-administered with live bacteria *in vitro*, CWA indirectly suppressed the production of pro-inflammatory mediators through phosphorylation of STAT-6, while inhibiting STAT-1 and NF-κB pathways. The resulting M2-like CD206^+^ macrophages displayed higher expression of M2 anti-inflammatory cytokines such as IL-4, IL-10, and TGF-β, but other crucial cellular functions such as phagocytosis were unchanged. CWA was also evaluated as a treatment for diarrhea in weaned piglets (Yi et al., 2016). Diarrheal diseases are a leading cause of death in young pigs and are usually associated with the proliferation of enterotoxigenic *E. coli* and heightened intestinal inflammation (Rhouma et al., 2017). CWA administration attenuated diarrhea and reduced levels of systemic and jejunal inflammation in piglets, similar to that observed using the fluoroquinolone antibiotic, enrofloxacin. However, only CWA treatment improved intestinal morphology, integrity and barrier functions, as well as microbial composition and metabolism (Yi et al., 2016).

Recently, Chen Y. et al. (2017) characterized the activities of six novel alligator cathelicidin peptides (As-CATH1-6). In general, As-CATH1-6 differed in their *in vitro* activity profiles and *in vivo* activities in murine peritonitis models of *E. coli* and *S. aureus* infection. Interestingly, the efficacy of these peptides in the murine models was related to their ability to recruit immune cells to the site of infection rather than assessed *in vitro* activities. Thus, peptides As-CATH4-6 displayed good antimicrobial, antibiofilm, and LPS-neutralization activities, while also suppressing production of pro-inflammatory mediators (NO, IL-6, IL-1β, and TNF-α) from LPS-stimulated murine peritoneal macrophages in contrast to the limited *in vitro* activities of As-CATH1-3. Nevertheless, when evaluated *in vivo*, As-CATH2-6 were all able to recruit neutrophils, monocytes, and macrophages to the infection site and protected mice against bacterial infection to varying degrees. Conversely, As-CATH-1 only moderately recruited macrophages and showed no protective effect *in vivo* (Chen Y. et al., 2017), suggesting a minor role for this HDP in preventing bacterial infections in alligators. Although the underlying reasons for these differences in efficacies among As-CATH2-6 were not further explored, these results highlight the importance of immune modulation in promoting the *in vivo* anti-infective activity of crocodylian cathelicidins.

In general, cathelicidins from a range of vertebrate species have displayed strong immunomodulatory effects including induction of chemokines and suppression of pro-inflammatory mediators that are induced in response to microbial signature molecules. Secondary to their role as immune modulators, cathelicidins reduce bacterial load in clinically relevant infection models. Cathelicidins also promote wound healing, cell recruitment and differentiation, and production of anti-inflammatory cytokines depending on their amino acid sequence. This functional plasticity likely contributes to the overall *in vivo* efficacy of these peptides. Thus, improving our understanding of the amino acid sequence requirements that control these biological functions is critical to decipher their role in preventing infections and maintaining overall health.

## Cathelicidins in Autoimmunity

Beyond their involvement in innate immunity, recent research has unveiled additional pathological roles for cathelicidins in auto-immune diseases and, in some instances, they have even been implicated as self-antigens. A common feature of immune-mediated chronic inflammation is an imbalance of cytokine and chemokine levels (Chen X. et al., 2018) and an imbalance in host levels of cathelicidin and other HDPs can potentially influence the concentrations of these inflammatory mediators. As our understanding of cellular and molecular mechanisms underlying HDP activities improves, their roles in various disease conditions are beginning to emerge.

Psoriasis is a common skin condition that afflicts an estimated 7.4 million adults in the United States alone (Rachakonda et al., 2014). Incidence rates vary from 1 to 3% based on age and geographical location and known risk factors include environmental stressors like smoking and a family history of psoriasis (Parisi et al., 2013). As an autoimmune condition, psoriasis is characterized by an excessive differentiation and maturation of T cells and DCs. Persistent epidermal inflammation and keratinocyte expansion coupled with reduced rates of cellular apoptosis results in scaly plaque formation on the skin surface. While the exact cause of psoriasis is unknown, this condition is associated with increased levels of LL-37 in the epidermis, which triggers the rapid recruitment and activation of neutrophils and T lymphocytes, likely contributing to a chronic inflammatory response encompassing innate and adaptive immunity (Schön, 2019). It was originally suggested that LL-37 protects RNA against degradation by forming complexes that induce translocation and activation of the TLR-7/8 and TLR-9 receptors (Ganguly et al., 2009). This was proposed to result in the enhancement of IFN-α and IFN-β release by pDCs, spurring the chronic inflammation typically associated with psoriatic epidermis.

Recent reports challenge this model, instead suggesting a role for neutrophils and neutrophil extracellular traps (NETs), rather than DCs, in psoriasis disease progression (Herster et al., 2020). NETs are DNA structures released from neutrophils with embedded cellular proteins and peptides, including HDPs, that are proposed to have various functions such as trapping of pathogens. Neutrophil RNA:LL-37, but not DNA:LL-37 complexes, activate signaling through human TLR-8 and murine TLR-13, to propagate the release of cytokines (e.g., TNF-α, IL-6, IL-8, IL-1β) and chemoattractants (e.g., IL-16, MIP-1β). The RNA:LL-37 complex further promotes inflammation by the enhanced activation of neutrophil-derived NETs that, in turn, propagate additional rounds of immune stimulation. Furthermore, NET-associated RNA can potentially trigger immune activation which might also play a part in other NET-associated diseases such as systemic lupus erythematosus (SLE).

The pathogenesis of SLE is poorly understood due to its heterogeneous nature, unexplained higher incidence rates in females, and unknown etiology (Rees et al., 2017). A common feature in SLE patients is that several innate immune functions are altered including functional disruptions of neutrophils, monocytes, macrophages, and DCs along with severe organ impairment. Interestingly, skin biopsies from SLE patients have reported slightly (1.3- to 1.5-fold) increased levels of LL-37, along with IFN-α and pDCs (Sun et al., 2011). Additionally, SLE-derived neutrophils exhibit an enhanced capacity to release self-DNA and peptides to form NET structures (Lande et al., 2011; Kahlenberg and Kaplan, 2013). NETs are stabilized and protected from degradation by LL-37 and activate signaling through TLR-9 in pDCs to produce type I IFNs. The NET-associated LL-37 complex leads to sustained inflammatory responses, therefore implicating LL-37 in SLE pathogenesis. Interestingly, auto-antibodies to LL-37 are also able to directly induce NET formation which, in turn, promotes higher levels of IFN-α and further propagates the chronic inflammation seen in SLE (Lande et al., 2011).

Rheumatoid arthritis (RA), is another autoimmune chronic inflammatory disease that afflicts more than a million people in the United States (Hunter et al., 2017). RA affects the synovial membrane in joints resulting in decay, disability and increased mortality (Aletaha and Smolen, 2018). Within the synovial membrane, human RA patients express higher LL-37 levels (assessed by immunohistochemistry) in both CD66b granulocytes and CD68 macrophages when compared to healthy donors (Hoffmann et al., 2013). The pristane-induced arthritis model in rats has been used to mimic human RA and reveals that rCRAMP (rat CRAMP) as well as defensin expression is enhanced in the synovial fluid compared to healthy controls (Hoffmann et al., 2013). These augmented levels (3- to 9-fold) of rat HDPs were found in the joints, blood, and lymphoid organs of pristane-treated rats and were associated with higher levels of IFN-γ and autoantibodies against rCRAMP, further strengthening the link between cathelicidins/HDPs and RA progression. Conversely, analogs of LL-37 have protective effects in another RA model, the murine collagen-induced arthritis model (Chow et al., 2014). Subcutaneous administration of IG-19, a 19 amino acid peptide derived from the internal sequence of LL-37, suppressed cellular infiltration of the collagen injection site and reduced inflammation markers when compared to untreated mice. IG-19 treatment was also found to improve the clinical score and reduce disease severity by alleviating the harmful inflammation associated with collagen-induced murine arthritis. Possibly increased expression of the parent peptide, LL-37, might represent a natural defense mechanism against RA.

LL-37 has also been implicated in atherosclerosis (Kahlenberg and Kaplan, 2013). Atherosclerosis is an inflammatory condition characterized by the deposition and accumulation of fatty and/or fibrous plaques on the artery walls. Cardiovascular diseases associated with atherosclerosis are common around the world and are a leading cause of heart attacks and strokes (Libby et al., 2019). LL-37 in atherosclerotic lesions, generated mainly by macrophages, was reported to enhance host immunity by promoting the secretion of cell surface adhesion molecules and chemokines (Edfeldt et al., 2006). Alternatively, it has been suggested that complexes of mitochondrial DNA and LL-37 escape autophagic degradation to amplify the inflammatory cascade and are key mediators of atherosclerotic plaque development (Zhang et al., 2015). Thus, there is evidence that abnormal levels of HDPs, like cathelicidins, might contribute to (or reflect) the development and pathogenesis of a range of immune-associated disorders. In the following section, we examine what is arguably the most serious case of immune system dysregulation, sepsis, and how HDP activities might combat this grave medical condition.

## Applications of Cathelicidins in Sepsis

Sepsis is a life-threatening condition caused by an abnormal and dysfunctional immune response to infection (Rittirsch et al., 2008). A recent global estimate reported 48.9 million cases of sepsis in 2017 and sepsis-associated mortality was found to be high, accounting for nearly 11 million (or 19.7% of all) deaths worldwide (Rudd et al., 2020). Despite its high global burden, a complete understanding of sepsis pathogenesis remains a daunting challenge in medicine. Sepsis was initially defined as blood poisoning, referring to the presence of bacteria in the blood, that led to an initial amplified inflammatory response, dubbed a “cytokine storm” (Chousterman et al., 2017), which a few days later was followed by a hypo-inflammatory phase (Lyle et al., 2014). A clinical consensus on the definition of sepsis was established in 1992 focusing on the initial uncontrolled inflammation and requiring a diagnosis of systemic inflammatory response syndrome. However, this failed to address the subsequent immunosuppressive characteristics of this syndrome that further complicated diagnosis and treatment, and that was associated with the greatest mortality (Lyle et al., 2014).

Transcriptomic studies have identified gene expression signatures associated with the progression of sepsis in patients (Pena et al., 2014). Whole blood, PBMCs, and even specific immune cells like neutrophils and monocytes have showcased both inflammation and immunosuppression/cellular reprogramming at early stages of sepsis in the clinic. Cellular reprogramming in early sepsis patients is related to repeated exposure of the immune system to bacterial endotoxin, thereby reducing the host response to microbial ligands, resulting in a situation dubbed “immune amnesia.” This type of immune dysfunction is implicated in high rates of ICU admission plus increased rates of organ failure that correlate with worse sepsis patient outcomes (Pena et al., 2014).

The majority of therapeutic candidates evaluated to date have sought to directly or indirectly dampen the initial pro-inflammatory immune response, but do not address the heterogeneity of sepsis pathogenesis, thereby resulting in ineffective therapies (Marshall, 2014). This high failure rate has led many to regard the development of sepsis treatment options as the “graveyard of biotech” (Riedemann et al., 2003). Since hyper-inflammation and immunosuppression appear to be acting in tandem, but manifest in a heterogeneous manner within the population, stratification of patients into phenotypically and mechanistically similar groups (endotypes) may well allow more targeted beneficial treatments and aid in future drug development (Van der Poll et al., 2017).

Perhaps unsurprisingly, the ability of natural HDPs to modulate immune responses has led researchers to examine whether these polypeptides play a role in sepsis progression and/or resolution. Interestingly, sepsis patients have been found to have ∼15-fold higher levels of defensins and lactoferrin HDPs, but not LL-37, relative to surgical controls (Berkestedt et al., 2010), suggesting an association of sepsis with an imbalance in natural HDP levels. Early work examining HDPs as treatments for sepsis used a murine model of endotoxemia where mice were given intravenous *E. coli* LPS. Exogenous application of human LL-37 protected mice from lethal sepsis by neutralizing endotoxin and suppressing the production of proinflammatory cytokines (Gough et al., 1996). LL-37 also protected against *S. aureus* septicemia (Bowdish et al., 2005b). Intriguingly, intratracheal instillation of LL-37 resulted in a reduction of TNF-α levels and significantly increased levels of chemoattractant MCP-1 in bronchoalveolar lavage fluid, suggesting that LL-37 specifically influenced the production of cytokines and chemokines (Scott et al., 2002). Using microarray studies, Scott et al. (2002) treated the murine macrophage cell line RAW 264.7 with LL-37 and found that upregulated genes fell into two broad categories associated with receptor binding and cellular communication. Both of these pathways promoted immune surveillance and the migration of immune cells, consistent with LL-37 having a protective role in sepsis progression. LL-37 and IB-367, a short analog of a the pig cathelicidin protegrin-1, were also evaluated in 3 rat models of sepsis: intraperitoneal delivery of LPS, peritonitis induced by live *E. coli*, and cecal ligation puncture (CLP) (Giacometti et al., 2003; Cirioni et al., 2006). Both LL-37 and IB-367 treatment in all three of these models dampened TLR signaling and reduced TNF-α secretion, thereby improving sepsis-associated inflammation. The CLP model in particular mimicked a multispecies intra-abdominal bacterial infection and has been found to best mirror the immune dysfunction in sepsis patients (Parker and Watkins, 2001). Lethality in the CLP model decreased from 100% in no treatment controls to 33.3% after treatment with LL-37 as well as the antibiotics polymyxin B and imipenem (Cirioni et al., 2006). Similarly, IB-367 led to 75% survival, as did piperacillin, when compared to the 100% lethality observed in the CLP control group (Giacometti et al., 2003). CLP lethality was completely overcome by the combination treatment of IB-367 and antibiotic, implying a combined effect of neutralizing endotoxin and limiting bacterial growth. *In vitro* and *in vivo* models have implicated certain mechanisms for LL-37 reduction of sepsis-induced injury, including decreased neutrophil infiltration, suppression of endothelial cell apoptosis, subdued inflammatory signal, and enhanced wound healing (Suzuki et al., 2011; Koziel et al., 2014; Qin et al., 2019).

The application of HDPs *in vitro* and *in vivo* has unveiled a variety of beneficial immunomodulatory effects that impact systemic inflammation in the context of sepsis (Martin et al., 2015). However, clinical applications of HDPs and related immunomodulatory proteins to treat sepsis are very limited, and results have been mixed. One of the more extensively studied proteins related to innate immunity is that of 80 kDa iron-binding protein human lactoferrin (LF) and various peptide fragments derived therefrom (Sinha et al., 2013). Bovine LF (bLF; 77% homologous to human LF) supplementation was evaluated in a randomized, placebo-controlled trial and led to a reduced incidence of sepsis in very low-birth-weight (VLBW) neonates compared to the placebo group, while oral bLF prevented necrotizing colitis of VLBW human infants by increasing regulatory T-cell counts (Akin et al., 2014). Inspired by the restoration of the immune imbalance by LF in human neonatal sepsis, a large clinical trial of 2,203 participants across 37 neonatal units was initiated to evaluate the effect of enteral feedings of bLF on sepsis prevention; unfortunately, this failed to improve neonatal sepsis rates (Griffiths et al., 2019). Similarly, LF derivatives like talactoferrin (Guntupalli et al., 2013) and the N-terminal 11 amino acids of LF, hLF1-11 (Martin et al., 2015), exhibited promising efficacy in animal models of sepsis, but failed to progress as successful sepsis therapeutics.

Alternative therapies are needed to improve sepsis patient outcomes and there is some evidence that the immunomodulatory features of HDPs and cathelicidins might prove beneficial in this regard. Monocytes and macrophages from septic patients exert immunosuppressive characteristics (Venet and Monneret, 2018) and share many commonalities with M2 macrophages (Pena et al., 2011). A novel method to restore immune balance has been proposed to promote an intermediate (M1-M2) macrophage phenotype using a modified synthetic bovine cathelicidin bactenecin known as innate defense regulator (IDR)-1018 (Pena et al., 2013). Specifically, maturation of primary blood monocytes in the presence of IDR-1018 altered the release of certain M1 cytokines (e.g., TNF-α, COX2, IP-10) while also enhancing the anti-inflammatory features of M2 macrophages (e.g., IL-10, CCL-22 but not TGF-β) to promote a return to a balanced immune response. Harnessing this type of macrophage reprogramming, in conjunction with the other immunomodulatory properties of synthetic HDPs, may allow beneficial manipulation of inflammatory responses in conditions such as sepsis.

## Synthetic HDPs and Clinical Applications

To demonstrate that small synthetic peptides with desirable activity profiles can be derived from natural HDPs, one needs to look no further than several LL-37 fragments that are naturally generated through the action of proteases found throughout the body (Table 1). LL-37 is released from the hCAP18 precursor protein through the action of proteinase 3 in the blood (Sørensen et al., 2001) or kallikrein 5 in the skin (Yamasaki et al., 2006). Interestingly, further proteolytic processing at the skin surface by serine proteases generates LL-37 fragments with enhanced antibacterial activity in dilute medium toward various skin pathogens such as *S. aureus* and *C. albicans* (Murakami et al., 2004; Yamasaki et al., 2006). Several studies have characterized various fragments of LL-37 primarily for their antimicrobial activity (Kanthawong et al., 2012) but also for their antibiofilm, antiviral, spermicidal, and immunomodulatory functions (Tripathi et al., 2015). These examples of synthetic cathelicidin-derived peptides do not even begin to scratch the surface of the many studies that manipulate the amino acid sequence of LL-37 and various peptide fragments to rationally design derivatives with enhanced biological activities (e.g., Chen et al., 2019).

**TABLE 1:** Naturally produced LL-37 fragments and their processing enzymes.

Peptide	Sequence	Processing enzyme^a^	References
LL-37	LLGDFFRKSKEKIGKEFKRIVQRIKDFLRNLVPRTES	PR3, K5	Sørensen et al., 2001; Yamasaki et al., 2006
RK-31	RKSKEKIGKEFKRIVQRIKDFLRNLVPRTES	SSP, K7	Murakami et al., 2004; Yamasaki et al., 2006
KS-30	KSKEKIGKEFKRIVQRIKDFLRNLVPRTES	SSP, K5	Murakami et al., 2004; Yamasaki et al., 2006
KR-20	KRIVQRIKDFLRNLVPRTES	SSP, K7	Murakami et al., 2004; Yamasaki et al., 2006
KS-22	KSKEKIGKEFKRIVQRIKDFLR	K5	Yamasaki et al., 2006
LL-29	LLGDFFRKSKEKIGKEFKRIVQRIKDFLR	K5	Yamasaki et al., 2006

*^*a*^PR3, proteinase 3 from neutrophils; K5, Kallikrein 5; K7, Kallikrein 7; SSP, Sweat serine protease.*

The majority of studies aimed at optimizing synthetic HDPs have focused on improving their direct antibacterial effects. As such, efficacy has been determined using endpoints such as recovered bacterial counts in dilute medium to measure success. Likely because factors present within the host milieu confound the direct antimicrobial activity of many HDPs, most of the subsequent trials have failed to advance therapeutic peptides beyond the pre-clinical stage (Mansour et al., 2014). Interestingly, even though the LL-37 fragments generated by the action of the kallikrein proteases have increased antibacterial potency, their ability to stimulate IL-8 release from keratinocytes is lost, suggesting that the peptide sequence requirements that underlie antibacterial activity are independent of those promoting immunomodulatory activity (Murakami et al., 2004). This may be another reason why many synthetic HDPs in clinical trials have failed to demonstrate success, since peptides with optimal antimicrobial activity *in vitro* may lack the immunomodulatory effects that are important or essential for their *in vivo* efficacy (Haney et al., 2019a). Considering that the immunomodulatory roles of HDPs are increasingly appreciated as their major natural function *in vivo* (Hancock et al., 2016), specifically selecting for peptides with enhanced immunomodulatory activities may prove more fruitful for advancing synthetic HDPs toward clinical applications.

Currently, 36 peptides are in clinical (27 peptides) and preclinical (9 peptides) trials for their potential applications in the context of infectious disease and immune modulation (Koo and Seo, 2019). Among HDPs in clinical trials is LL-37 itself, that was evaluated as a potential treatment of venous leg ulcers (VLUs). VLUs are the most prevalent form of chronic wounds caused by venous insufficiency or arterial disease (i.e., malfunctioning circulatory valves) that lead to significant intravenous pressure, and inflammation (Chi and Raffetto, 2015). Acute skin wounding of normal healthy skin induces the expression of human hCAP18 by epidermal keratinocytes at the wound margin. However, hCAP18 mRNA is rapidly degraded and functional peptide is reduced in chronic skin wounds, such as those associated with VLUs (Heilborn et al., 2003). Reduced LL-37 levels correspondingly reduce the activation of leukocytes and inhibit inflammatory cascades that are important for clearing pathogens and promoting angiogenesis, thereby preventing venous leg ulcers from healing appropriately.

Topical administration of LL-37 at low doses was assessed in combination with compression therapy for treatment of venous leg ulcers and comorbid bacterial infections in a randomized, placebo-controlled, double-blind Phase IIa clinical trial (Grönberg et al., 2014). A significant improvement in wound healing was observed in patients treated with 0.5 or 1.6 mg/ml LL-37 compared to patients treated with placebo, whereas no improvement was seen for patients treated with 3.2 mg/ml LL-37. Indeed, patients treated with the highest concentration of LL-37 demonstrated increased inflammation at the wound site although only one of these cases was classified as severe. An ongoing Phase IIb clinical trial has been initiated to advance the peptide through the drug development pipeline (Rivas-Santiago et al., 2012), but the results of that clinical trial have not been published to date.

Although vitamin D_3_ and 1,25-dihydroxyvitamin D upregulate the expression of LL-37 and enhance wound healing in a keratinocyte model of diabetic foot ulcers *ex vivo* (Gonzalez-Curiel et al., 2014), endogenous levels of vitamin D_3_ do not correlate with healing of VLUs *in vivo* (Krejner et al., 2017). These contrasting results highlight the discrepant influence of vitamin D_3_ on LL-37 activity *in vitro* vs. *in vivo* and demonstrate the importance of considering the effects of physiological solutes in cell-culture based mechanistic characterization of HDPs. Furthermore, the rapid degradation of vitamin D_3_ might explain the failure of oral supplementation to improve diabetic wound healing in a randomized, placebo-controlled clinical trial (NCT03813927). In contrast, a recent randomized, double-blind, placebo-controlled trial in Bangladesh examined the effect of supplementation with vitamin D3 and 4-phenyl butyrate, both of which are potent inducers of LL-37, on recovery of adult tuberculosis patients aged 18–24. After 4 weeks of treatment, 71% of tuberculosis patients given both supplements (*p* = 0.001) and 61.3% of vitamin D3 supplemented patients (*p* = 0.032) were culture negative, compared to only 42.2% in the placebo-group (Mily et al., 2015). LL-37 levels were correspondingly increased and intracellular growth of *Mycobacterium tuberculosis* decreased, consistent with published animal model studies (Rivas-Santiago et al., 2013). Thus the induction of LL-37 represents a novel and exciting new strategy for the treatment of tuberculosis utilizing rather inexpensive inducers.

P60.4Ac is a 24-residue C-terminal truncated derivative of human LL-37 that was originally designed to inhibit inflammation associated with chronic sinusitis and other diseases of the upper respiratory tract (Nell et al., 2006). In a bronchial epithelial model of respiratory mucosa *in vitro*, P60.4Ac reduced LPS-induced TLR signaling and downstream activation of extracellular signal-related kinase Erk1/2, which in the context of upper respiratory infections is known to trigger damaging inflammatory responses (Nell et al., 2006). Excitingly, when administered as ear drops to patients suffering from chronic otitis media in a randomized, placebo-controlled Phase IIb clinical trial, P60.4Ac demonstrated efficacy in reducing bacterial load and neutrophil infiltration (Peek et al., 2009). In 2010, plans for a Phase III clinical trial were announced for this peptide, renamed as OP-145^1^ but the company making this announcement was sold and there is no indication the trial has proceeded. Nevertheless, formulated OP-145 continues to be studied preclinically for potential applications in the context of biomaterial-associated infections (De Breij et al., 2016).

Omiganan is a 12-residue derivative of the bovine cathelicidin, indolicidin, that has been in development as a topical antimicrobial compound for several years (Rubinchik et al., 2009). When applied as an antimicrobial, omiganan was unsuccessful in a Phase IIIb clinical trial for the treatment of catheter-related urinary tract infections due to failure to reach its primary endpoint (significant decrease in physician-determined infections) although it showed efficacy in its secondary endpoints of significantly decreased microbiologically confirmed infections and decreased tunnel infections, demonstrating its potential (Koo and Seo, 2019). More recently, formulation of omiganan (also known as CLS-001, MBI-226, and/or MX-226 across studies) as a topical gel revealed clinical promise as an immunomodulatory/anti-inflammatory treatment of rosacea, acne, vulvar intraepithelial neoplasia, and atopic dermatitis, according to preliminary results from Phase IIIa clinical trials^2^. Interestingly, little mechanistic data has been reported to elucidate its immunomodulatory influence on host cells. *In vitro*, omiganan has broad spectrum antimicrobial and antifungal activity against numerous resistant species including *S. aureus* and *Candida albicans*, respectively; however, proteases endogenous to the skin can cause deactivation of the peptide *in situ* (Ng et al., 2017). In contrast, the D-enantiomer of omiganan is metabolically stable toward skin proteases and is comparable to the L-enantiomer in terms of antibacterial and antifungal potency. Although further studies are needed to determine suitability of the D-enantiomeric peptide for *in vivo* use, preliminary studies suggest that the stereoisomer of omiganan remains a promising candidate for future clinical applications (Zapotoczna et al., 2017).

The possibility of extrinsically manipulating endogenous expression of *CAMP* for systemic and localized therapeutic benefit, as mentioned above for LL-37 in the case of tuberculosis, has also attracted significant attention in recent years (Brandwein et al., 2017). Several epidemiological studies have linked serum and/or tissue levels of LL-37 to clinical outcomes in seemingly unrelated diseases such as bacterial meningitis, rosacea, respiratory syncytial virus (RSV) bronchiolitis, and type II diabetes mellitus. In the context of bacterial meningitis, a substantial bacterial load in the membranes that surround the spinal cord and brain is associated with poor outcomes and cerebrospinal fluid with high levels of LL-37 was linked to reduced neurological damage and audiological sequelae, but not improved survival in children (Savonius et al., 2018). Rosacea is characterized as an erythematous pustular rash on the face with overexpressed LL-37 levels in tissues and serum of patients (Park et al., 2018). Interestingly, LL-37 expression in the skin of rosacea patients is abnormally high (Yamasaki et al., 2007) and it has been proposed that this leads to an overactive innate immune response that contributes to disease pathogenesis. Regarding viral infections, higher levels of serum LL-37 in human infants were associated with lower rates of RSV bronchiolitis, but the causality of LL-37 levels and disease severity lacks elucidation (Mansbach et al., 2017). Lastly, in the case of type II diabetes mellitus, decreased LL-37 levels are associated with lowered high-density lipoprotein (HDL) cholesterol, which in turn diminishes the buildup of atherosclerotic plaque load and therefore, cardiovascular damage (Meguro et al., 2014). Unfortunately, attempts to modulate *CAMP* levels at the transcriptional and protein levels have demonstrated variable success in the clinic (Mily et al., 2013; Mily et al., 2015). This may be related to several issues including how inducers are delivered, endogenous levels of LL-37 in patients and/or proteolytic degradation *in situ*.

## Novel Formulations for Peptide Delivery

To date, the inability to translate animal model data into clinical applications for many peptide-based drugs might be due to a range of factors including a short half-life, a tendency for peptides to aggregate, non-specific toxicity at high concentrations as well as confounding physiological factors *in vivo.* These factors need to be considered when advancing a novel peptide through clinical trials and many of them can be addressed through the use of drug formulation strategies (Eckert, 2011), or circumvented by incorporating active peptides directly into biomedical devices (Riool et al., 2017). For example, peptides can be immobilized into fibrous scaffolds, called electrospun nanofibers, to create functional wound dressings (Amariei et al., 2018). Another strategy involving the synthetic antimicrobial peptide, HHC-36, incorporated this peptide into titanium coatings to control the release of peptide from orthopedic implants and prevent the development of bacterial biofilms on implant surfaces (Kazemzadeh-Narbat et al., 2013). A similar approach sought to prevent bacterial attachment on implant surfaces by coating medical devices with a polymer brush decorated with covalently-attached synthetic AMPs while maintaining their bactericidal effects against Gram-positive and Gram-negative bacteria (Yu et al., 2015). Although some peptide delivery methods have been evaluated in the clinical trials described above, many are still being assessed in preclinical studies. Oftentimes, *in vitro* efficacy testing of nascent and formulated peptides hinges on isolation of primary cells from human volunteers or working with immortalized cell lines. From there, testing in animal models allows efficacy to be determined under physiological conditions. In these models, peptides can be delivered locally to a site of infection or inflammation in a variety of ways (e.g., via implanted catheters or adsorption to other implanted biomedical plastics) but most have been best studied following topical cream formulation (Easton et al., 2009). The application of novel formulations in animal models that best mimic these scenarios is crucial to bridging the gap between pre-clinical and clinical development of cathelicidins.

The incorporation of synthetic HDPs with potent immunomodulatory properties into vaccine adjuvants is an attractive area of research with the goal of enhancing the adaptive immune response elicited by existing, clinically-approved therapies to garner greater immune protection (Nicholls et al., 2010). The feasibility of such an approach was demonstrated with a polyphosphazene microparticle formulation incorporating IDR-1002 with the TLR-9 agonist, CpG oligodeoxynucleotide, leading to enhanced protection in a murine model of whooping cough (Polewicz et al., 2013). Compared to microparticles alone, the peptide formulated adjuvant stimulated enhanced immune activity against *Bordetella pertussis* by upregulating the secretion of numerous chemokines and cytokines including MCP-1 and TNF-α as well as dampening the secretion of anti-inflammatory IL-10.

Several other peptide formulation strategies have focused on prolonging the release of active compound to provide a sustained therapeutic effect, or to prevent some of the negative effects of administering high concentrations of peptides (e.g., aggregation and/or toxicity). Cathelicidin-BF is a peptide isolated from the venom of the banded krait snake, *Bungarus fasciatus*, that has proven amenable to microparticle formulation. Formulation of this peptide in poly(D,L-lactide-co-glycolide) (PLGA) microspheres allowed for the slow release of cathelicidin-BF *in vitro* for more than 15 days. Importantly, the microspheres prevented peptide degradation while still maintaining antimicrobial efficacy against *E. coli*, *Shigella dysenteriae*, and *Salmonella typhi* (Bao et al., 2018). In addition, using a murine model of *P. aeruginosa* pneumonia, intravenous pre-treatment of formulated cathelicidin-BF activated the immune response to improve antibacterial functions while enhancing macrophage clearance and dampening inflammation via obstruction of the NF-κB signaling cascade (Liu et al., 2018). Cathelicidin-BF has also been conjugated to PLGA polymers, resulting in even slower release (up to 30 days) while exhibiting minimal toxicity toward eukaryotic cells as well as low hemolysis (Schlosser et al., 2008). The slow release of co-encapsulated PLGA microparticles resulted in enhanced DC uptake and activation of cytotoxic T cells and furthermore, protected against vaccinia virus *in vivo*. Lastly, IDR-1018 peptide formulated with derivatized hyperbranched polyglycerols (dHPG) provided a novel strategy to minimize peptide aggregation and toxicity (Haney et al., 2019b). Administration of IDR-1018 formulated with dHPG sustained *in vitro* immunomodulatory features toward human PBMCs stimulated with LPS and a human bronchial epithelial cell line stimulated with polyinosinic:polycytidylic acid, modulating TLR-4 or TLR-3 signaling, respectively.

Liposomal/micellar formulations serve to stabilize peptides in *in vitro* and *in vivo* models. While cellular toxicity of peptides is often diminished when formulating with lipids, activity is often preserved as seen in studies examining methicillin resistant *S. aureus* skin infections (Kumar et al., 2019), herpes simplex virus 1 infection (Ron-Doitch et al., 2016), and has even shown effectiveness as a tumor delivery system (Zhang et al., 2016). Formulation of a synthetic antibiofilm peptide, DJK-5, in nanogels composed of octenyl succinic anhydride-modified hyaluronic acid decreased cutaneous toxicity 4-fold when compared to DJK-5 alone (Kłodzińska et al., 2019). Importantly, DJK-5 nanogels maintained their efficacy against *P. aeruginosa* LESB58-generated murine abscesses. Thus, formulation strategies that promote peptide stability while also mitigating potentially negative effects related to peptide toxicity and aggregation hold tremendous promise to aid in clearing the final hurdle to advancing synthetic HDPs to the clinic.

## Conclusion and Future Directions

HDPs are distributed across a broad range of vertebrate and invertebrate species. The cathelicidin subgroup in vertebrates in particular contributes to the complex signaling mechanisms that are associated with the innate immune response as well as various inflammatory processes. Since HDPs are able to bind to and modulate signaling through TLRs and other extracellular and intracellular receptors, they massively alter gene expression in cells and can influence downstream effects in the cell to modulate the immune response. This leads to the alteration of cytokine and chemokine levels, depending on other underlying immune stimuli, promoting chemotaxis and cellular proliferation, suppressing inflammation, and providing a link between the innate and adaptive immune systems. Rather than complete suppression of cellular responses and signaling, HDPs, and cathelicidins in particular, enforce a more balanced immune response. Unsurprisingly, atypical cathelicidin expression has been shown to exhibit contradictory and harmful effects as evidenced by reports of cathelicidins acting as self-antigens as well as being implicated in the pathogenesis of complex autoimmune diseases. In the context of persistent dysfunctional inflammation in sepsis, the ability of HDPs to modulate cellular differentiation and alter inflammatory signaling pathways may prove to be beneficial for disease resolution. To harness the useful characteristics of peptides under physiological conditions, the choice of an appropriate formulation for synthetic HDPs is of great importance. Formulation lends itself to improved peptide stability and thus sustained functionalities, enabling HDPs to impact on their diverse signaling pathways in the context of inflammatory and infectious diseases. As we seek new therapies based on natural HDPs, a strategy that seeks to maximize the host immune response is potentially the best path forward toward advancing synthetic HDPs toward clinical applications.

## Author Contributions

All authors were involved in writing and editing the manuscript.

## Conflict of Interest

RH and EH have developed peptides, related to the ones discussed here, for commercial application, assigned these to the employer the University of British Columbia and licensed these to ABT therapeutics Inc., a Victoria company owned in part by RH with EH as a minor shareholder. The remaining authors declare that the research was conducted in the absence of any commercial or financial relationships that could be construed as a potential conflict of interest.

## Footnotes

1 http://www.octoplus.nl

2 http://www.mallinckrodt.com/

## References

[B1] Agier J. Brzezińska-Błaszczyk E. Zelechowska P. Wiktorska M. Pietrzak J. Rózalska S. (2018). Cathelicidin LL-37 affects surface and intracellular toll-like receptor expression in tissue mast cells. *J. Immunol. Res.* 2018:7357162. 10.1155/2018/7357162 29670923 PMC5836302

[B2] Akin I. M. Atasay B. Dogu F. Okulu E. Arsan S. Karatas H. D. (2014). Oral lactoferrin to prevent nosocomial sepsis and necrotizing enterocolitis of premature neonates and effect on T-regulatory cells. *Am. J. Perinatol.* 31 1111–1120. 10.1055/s-0034-1371704 24839144

[B3] Aletaha D. Smolen J. S. (2018). Diagnosis and management of rheumatoid arthritis: a review. *JAMA* 320 1360–1372. 10.1001/jama.2018.13103 30285183

[B4] Amariei G. Kokol V. Boltes K. Letón P. Rosal R. (2018). Incorporation of antimicrobial peptides on electrospun nanofibres for biomedical applications. *RSC Adv.* 8 28013–28023. 10.1039/C8RA03861A PMC9083935 35542741

[B5] Andersson E. Sørensen O. E. Frohm B. Borregaard N. Egesten A. Malm J. (2002). Isolation of human cationic antimicrobial protein-18 from seminal plasma and its association with prostasomes. *Hum. Reprod.* 17 2529–2534. 10.1093/humrep/17.10.2529 12351523

[B6] Bao Y. Wang S. Li H. Wang Y. Chen H. Yuan M. (2018). Characterization, stability and biological activity in vitro of cathelicidin-BF-30 loaded 4-Arm star-shaped PEG-PLGA microspheres. *Molecules* 23:497. 10.3390/molecules23020497 29473887 PMC6017235

[B7] Barlow P. G. Beaumont P. E. Cosseau C. Mackellar A. Wilkinson T. S. Hancock R. E. W. (2010). The human cathelicidin LL-37 preferentially promotes apoptosis of infected airway epithelium. *Am. J. Respir Cell. Mol. Biol.* 43 692–702. 10.1165/rcmb.2009-0250OC 20097832 PMC2993089

[B8] Berkestedt I. Herwald H. Ljunggren L. Nelson A. Bodelsson M. (2010). Elevated plasma levels of antimicrobial polypeptides in patients with severe sepsis. *J. Innate Immun.* 2 478–482. 10.1159/000317036 20571257

[B9] Beutler B. A. (2009). TLRs and innate immunity. *Blood* 113 1399–1407. 10.1182/blood-2008-07-019307 18757776 PMC2644070

[B10] Boman H. G. (2003). Antibacterial peptides: basic facts and emerging concepts. *J. Int. Med.* 254 197–215. 10.1046/j.1365-2796.2003.01228.x 12930229

[B11] Bowdish D. M. E. Davidson D. J. Scott M. G. Hancock R. E. W. (2005a). Immunomodulatory activities of small host defense peptides. *Antimicrob. Agents Chemother.* 49 1727–1732. 10.1128/AAC.49.5.1727-1732.2005 15855488 PMC1087655

[B12] Bowdish D. M. E. Davidson D. J. Lau Y. E. Lee K. Scott M. G. Hancock R. E. W. (2005b). Impact of LL-37 on anti-infective immunity. *J. Leukoc. Biol.* 77 451–459. 10.1189/jlb.0704380 15569695

[B13] Brandwein M. Bentwich Z. Steinberg D. (2017). Endogenous antimicrobial peptide expression in response to bacterial epidermal colonization. *Front. Immunol.* 8:1637. 10.3389/fimmu.2017.01637 29230218 PMC5711782

[B14] Cai S. Qiao X. Feng L. Shi N. Wang H. Yang H. (2018). Python cathelicidin CATHPb1 protects against multidrug-resistant staphylococcal infections by antimicrobial-immunomodulatory duality. *J. Med. Chem.* 61 2075–2086. 10.1021/acs.jmedchem.8b00036 29466000

[B15] Cao X. Wang Y. Wu C. Li X. Fu Z. Yang M. (2018). Cathelicidin-OA1, a novel antioxidant peptide identified from an amphibian, accelerates skin wound healing. *Sci. Rep.* 8:943. 10.1038/s41598-018-19486-9 29343843 PMC5772731

[B16] Chen J. Vitetta L. (2020). The role of butyrate in attenuating pathobiont-induced hyperinflammation. *Immune Netw.* 20:e15. 10.4110/in.2020.20.e15 32395367 PMC7192831

[B17] Chen S. Lu Z. Wang F. Wang Y. (2018). Cathelicidin-WA polarizes *E. coli K*88-induced M1 macrophage to M2-like macrophage in RAW264.7 cells. *Int. Immunopharmacol.* 54 52–59. 10.1016/j.intimp.2017.10.013 29101873

[B18] Chen X. Zou X. Qi G. Tang Y. Guo Y. Si J. (2018). Roles and mechanisms of human cathelicidin LL-37 in cancer. *Cell Physiol. Biochem.* 47 1060–1073. 10.1159/000490183 29843147

[B19] Chen Y. Cai S. Qiao X. Wu M. Guo Z.-L. Wang R. (2017). As-CATH1-6, novel cathelicidins with potent antimicrobial and immunomodulatory properties from *Alligator sinensis*, play pivotal roles in host antimicrobial immune responses. *Biochem. J.* 474 2861–2885. 10.1042/BCJ20170334 28798159

[B20] Chen Z. Yang G. Lu S. Chen D. Fan S. Xu J. (2019). Design and antimicrobial activities of LL-37 derivatives inhibiting the formation of *Streptococcus mutans* biofilm. *Chem. Biol. Drug Des.* 93 1175–1185. 10.1111/cbdd.13419 30635992

[B21] Cheng Y. Prickett M. D. Gutowska W. Kuo R. Belov K. Burt D. W. (2015). Evolution of the avian β-defensin and cathelicidin genes. *BMC Evol. Biol.* 15:188. 10.1186/s12862-015-0465-3 26373713 PMC4571063

[B22] Chi Y. W. Raffetto J. D. (2015). Venous leg ulceration pathophysiology and evidence based treatment. *Vasc. Med.* 20 168–181. 10.1177/1358863X14568677 25832604

[B23] Chousterman B. G. Swirski F. K. Weber G. F. (2017). Cytokine storm and sepsis disease pathogenesis. *Semin. Immunopathol.* 39 517–528. 10.1007/s00281-017-0639-8 28555385

[B24] Chow L. N. Y. Choi K-Y (Grace) Piyadasa H. Bossert M. Uzonna J. Klonisch T. (2014). Human cathelicidin LL-37-derived peptide IG-19 confers protection in a murine model of collagen-induced arthritis. *Mol. Immunol.* 57 86–92. 10.1016/j.molimm.2013.08.011 24091294

[B25] Cirioni O. Giacometti A. Ghiselli R. Bergnach C. Orlando F. Silvestri C. (2006). LL-37 protects rats against lethal sepsis caused by gram-negative bacteria. *Antimicrob. Agents Chemother.* 50 1672–1679. 10.1128/AAC.50.5.1672-1679.2006 16641434 PMC1472226

[B26] Clarke B. T. (1997). The natural history of amphibian skin secretions, their normal functioning and potential medical applications. *Biol. Rev.* 72 365–379. 10.1111/j.1469-185X.1997.tb00018.x 9336100

[B27] Coorens M. Scheenstra M. R. Veldhuizen E. J. A. A. Haagsman H. P. (2017). Interspecies cathelicidin comparison reveals divergence in antimicrobial activity, TLR modulation, chemokine induction and regulation of phagocytosis. *Sci. Rep.* 7 1–11. 10.1038/srep40874 28102367 PMC5244392

[B28] Dalla Valle L. Benato F. Paccanaro M. C. Alibardi L. (2013). Bioinformatic and molecular characterization of cathelicidin-like peptides isolated from the green lizard *Anolis carolinensis* (Reptilia: Lepidosauria: Iguanidae). *Ital. J. Zool.* 80 177–186. 10.1080/11250003.2013.783632

[B29] Davidson D. J. Currie A. J. Reid G. S. D. MacDonald K. L. Ma R. C. Speert D. P. (2004). The cationic antimicrobial peptide LL-37 modulates dendritic cell differentiation and dendritic cell-induced T cell polarization. *J. Immunol.* 172 1146–1156. 10.4049/jimmunol.172.2.1146 14707090

[B30] De Barros E. Gonçalves R. M. Cardoso M. H. Santos N. C. Franco O. L. Cândido E. S. (2019). Snake venom cathelicidins as natural antimicrobial peptides. *Front. Pharmacol.* 10:1415. 10.3389/fphar.2019.01415 31849667 PMC6895205

[B31] De Breij A. Riool M. Kwakman P. H. S. De Boer L. Cordfunke R. A. Drijfhout J. W. (2016). Prevention of *Staphylococcus aureus* biomaterial-associated infections using a polymer-lipid coating containing the antimicrobial peptide OP-145. *J. Control Release* 222 1–8. 10.1016/j.jconrel.2015.12.003 26658071

[B32] Easton D. M. Nijnik A. Mayer M. L. Hancock R. E. W. (2009). Potential of immunomodulatory host defense peptides as novel anti-infectives. *Trends Biotechnol.* 27 582–590. 10.1016/j.tibtech.2009.07.004 19683819 PMC7114281

[B33] Eckert R. (2011). Road to clinical efficacy: challenges and novel strategies for antimicrobial peptide development. *Future Microbiol.* 6 635–651. 10.2217/fmb.11.27 21707311

[B34] Edfeldt K. Agerberth B. Rottenberg M. E. Gudmundsson G. H. Wang X. B. Mandal K. (2006). Involvement of the antimicrobial peptide LL-37 in human atherosclerosis. *Arterioscler Thromb. Vasc. Biol.* 26 1551–1557. 10.1161/01.ATV.0000223901.08459.57 16645154

[B35] Elloumi H. Z. Holland S. M. (2008). Complex regulation of human cathelicidin gene expression: novel splice variants and 5’UTR negative regulatory element. *Mol. Immunol.* 45 204–217. 10.1016/j.molimm.2007.04.023 17709140 PMC2121615

[B36] Fagerberg L. Hallstrom B. M. Oksvold P. Kampf C. Djureinovic D. Odeberg J. (2014). Analysis of the human tissue-specific expression by genome-wide integration of transcriptomics and antibody-based proteomics. *Mol. Cell Proteom.* 13 397–406. 10.1074/mcp.M113.035600 24309898 PMC3916642

[B37] Fjell C. D. Hiss J. A. Hancock R. E. W. Schneider G. (2012). Designing antimicrobial peptides: form follows function. *Nat. Rev. Drug Discov.* 11 37–51. 10.1038/nrd3591 22173434

[B38] Fruitwala S. El-Naccache D. W. Chang T. L. (2019). Multifaceted immune functions of human defensins and underlying mechanisms. *Semin Cell Dev. Biol.* 88 163–172. 10.1016/j.semcdb.2018.02.023 29501617 PMC6485945

[B39] Ganguly D. Chamilos G. Lande R. Gregorio J. Meller S. Facchinetti V. (2009). Self-RNA-antimicrobial peptide complexes activate human dendritic cells through TLR7 and TLR8. *J. Exp. Med.* 206 1983–1994. 10.1084/jem.20090480 19703986 PMC2737167

[B40] Giacometti A. Cirioni O. Ghiselli R. Mocchegiani F. Viticchi C. Orlando F. (2003). Antiendotoxin activity of protegrin analog IB-367 alone or in combination with piperacillin in different animal models of septic shock. *Peptides* 24 1747–1752. 10.1016/j.peptides.2003.07.027 15019206

[B41] Gombart A. F. Borregaard N. Koeffler H. P. (2005). Human cathelicidin antimicrobial peptide (CAMP) gene is a direct target of the vitamin D receptor and is strongly up-regulated in myeloid cells by 1,25-dihydroxyvitamin D3. *FASEB J.* 19 1067–1077. 10.1096/fj.04-3284com 15985530

[B42] Gonzalez-Curiel I. Trujillo V. Montoya-Rosales A. Rincon K. Rivas-Calderon B. De Haro-Acosta J. (2014). 1,25-dihydroxyvitamin D3 induces LL-37 and HBD-2 production in keratinocytes from diabetic foot ulcers promoting wound healing: an in vitro model. *PLoS One* 9:e111355. 10.1371/journal.pone.0111355 25337708 PMC4206472

[B43] Gough M. Hancock R. E. Kelly N. M. (1996). Antiendotoxin activity of cationic peptide antimicrobial agents. *Infect. Immun.* 64 4922–4927. 10.1128/iai.64.12.4922-4927.1996 8945527 PMC174469

[B44] Griffiths J. Jenkins P. Vargova M. Bowler U. Juszczak E. King A. (2019). Enteral lactoferrin supplementation for very preterm infants: a randomised placebo-controlled trial. *Lancet* 393 423–433. 10.1016/S0140-6736(18)32221-9 30635141 PMC6356450

[B45] Grönberg A. Mahlapuu M. Ståhle M. Whately-Smith C. Rollman O. (2014). Treatment with LL-37 is safe and effective in enhancing healing of hard-to-heal venous leg ulcers: a randomized, placebo-controlled clinical trial. *Wound Repair. Regen.* 22 613–621. 10.1111/wrr.12211 25041740

[B46] Guntupalli K. Dean N. Morris P. E. Bandi V. Margolis B. Rivers E. (2013). A phase 2 randomized, double-blind, placebo-controlled study of the safety and efficacy of talactoferrin in patients with severe sepsis. *Crit. Care Med.* 41 706–716. 10.1097/CCM.0b013e3182741551 23425819

[B47] Hahn M. W. Demuth J. P. Han S.-G. (2007). Accelerated rate of gene gain and loss in primates. *Genetics* 177 1941–1949. 10.1534/genetics.107.080077 17947411 PMC2147951

[B48] Hancock R. E. W. Haney E. F. Gill E. E. (2016). The immunology of host defence peptides: beyond antimicrobial activity. *Nat. Rev. Immunol.* 16 321–334. 10.1038/nri.2016.29 27087664

[B49] Haney E. F. Straus S. K. Hancock R. E. W. (2019a). Reassessing the host defense peptide landscape. *Front. Chem.* 7:43. 10.3389/fchem.2019.00043 30778385 PMC6369191

[B50] Haney E. F. Wuerth K. C. Rahanjam N. Safaei Nikouei N. Ghassemi A. Alizadeh Noghani M. (2019b). Identification of an IDR peptide formulation candidate that prevents peptide aggregation and retains immunomodulatory activity. *Pept. Sci.* 111:e24077 10.1002/pep2.24077

[B51] Heilborn J. D. Nilsson M. F. Kratz G. Weber G. Sørensen O. E. Borregaard N. (2003). The cathelicidin anti-microbial peptide LL-37 is involved in re-epithelialization of human skin wounds and is lacking in chronic ulcer epithelium. *J. Invest. Dermatol.* 120 379–389. 10.1046/j.1523-1747.2003.12069.x 12603850

[B52] Herster F. Bittner Z. Archer N. K. Dickhöfer S. Eisel D. Eigenbrod T. (2020). Neutrophil extracellular trap-associated RNA and LL37 enable self-amplifying inflammation in psoriasis. *Nat. Commun.* 11:105. 10.1038/s41467-019-13756-4 31913271 PMC6949246

[B53] Hilchie A. L. Wuerth K. Hancock R. E. W. (2013). Immune modulation by multifaceted cationic host defense (antimicrobial) peptides. *Nat. Chem. Biol.* 9 761–768. 10.1038/nchembio.1393 24231617

[B54] Hoffmann M. H. Bruns H. Bac̈kdahl L. Neregar̊d P. Niederreiter B. Herrmann M. (2013). The cathelicidins LL-37 and rCRAMP are associated with pathogenic events of arthritis in humans and rats. *Ann. Rheum. Dis.* 72 1239–1248. 10.1136/annrheumdis-2012-202218 23172753

[B55] Horibe K. Nakamichi Y. Uehara S. Nakamura M. Koide M. Kobayashi Y. (2013). Roles of cathelicidin-related antimicrobial peptide in murine osteoclastogenesis. *Immunology* 140 344–351. 10.1111/imm.12146 23826736 PMC3800439

[B56] Huang L. C. Reins R. Y. Gallo R. L. McDermott A. M. (2007). Cathelicidin-Deficient (Cnlp-/-) Mice Show Increased Susceptibility to *Pseudomonas aeruginosa* Keratitis. *Invest. Ophthalmol. Vis. Sci.* 48 4498–4508. 10.1167/iovs.07-0274 17898271 PMC4234056

[B57] Hunter T. M. Boytsov N. N. Zhang X. Schroeder K. Michaud K. Araujo A. B. (2017). Prevalence of rheumatoid arthritis in the United States adult population in healthcare claims databases, 2004–2014. *Rheumatol. Int.* 37 1551–1557. 10.1007/s00296-017-3726-1 28455559

[B58] Inomata M. Horie T. Into T. (2019). Effect of the antimicrobial peptide LL-37 on gene expression of chemokines and 29 toll-like receptor-associated proteins in human gingival fibroblasts under stimulation with *Porphyromonas gingivalis* lipopolysaccharide. *Probiot. Antimicrob Proteins* 12 64–72. 10.1007/s12602-019-09600-2 31686299

[B59] Ji P. Zhou Y. Yang Y. Wu J. Zhou H. Quan W. (2019). Myeloid cell-derived LL-37 promotes lung cancer growth by activating Wnt/β-catenin signaling. *Theranostics* 9 2209–2223. 10.7150/thno.30726 31149039 PMC6531301

[B60] Kahlenberg J. M. Kaplan M. J. (2013). Little peptide, big effects: the role of LL-37 in inflammation and autoimmune disease. *J. Immunol.* 191 4895–4901. 10.4049/jimmunol.1302005 24185823 PMC3836506

[B61] Kai-Larsen Y. Agerberth B. (2008). The role of the multifunctional peptide LL-37 in host defense. *Front. Biosci.* 13:3760–3767. 10.2741/2964 18508470

[B62] Kandler K. Shaykhiev R. Kleemann P. Klescz F. Lohoff M. Vogelmeier C. (2006). The anti-microbial peptide LL-37 inhibits the activation of dendritic cells by TLR ligands. *Int. Immunol.* 18 1729–1736. 10.1093/intimm/dxl107 17041145

[B63] Kanthawong S. Bolscher J. G. M. Veerman E. C. I. van Marle J. de Soet H. J. J. Nazmi K. (2012). Antimicrobial and antibiofilm activity of LL-37 and its truncated variants against *Burkholderia pseudomallei*. *Int. J. Antimicrob. Agents* 39 39–44. 10.1016/j.ijantimicag.2011.09.010 22005071

[B64] Kazemzadeh-Narbat M. Lai B. F. L. Ding C. Kizhakkedathu J. N. Hancock R. E. W. Wang R. (2013). Multilayered coating on titanium for controlled release of antimicrobial peptides for the prevention of implant-associated infections. *Biomaterials* 34 5969–5977. 10.1016/j.biomaterials.2013.04.036 23680363

[B65] Kim D. Soundrarajan N. Lee J. Cho H.-S. Choi M. Cha S.-Y. (2017). Genomewide analysis of the antimicrobial peptides in *Python bivittatus* and characterization of cathelicidins with potent antimicrobial activity and low cytotoxicity. *Antimicrob. Agents Chemother.* 61 1–12. 10.1128/AAC.00530-17 28630199 PMC5571288

[B66] Kłodzińska S. N. Pletzer D. Rahanjam N. Rades T. Hancock R. E. W. Nielsen H. M. (2019). Hyaluronic acid-based nanogels improve in vivo compatibility of the anti-biofilm peptide DJK-5. *Nanomed. Nanotechnol. Biol. Med.* 20:102022. 10.1016/j.nano.2019.102022 31170510

[B67] Koo H. B. Seo J. (2019). Antimicrobial peptides under clinical investigation. *Pept. Sci.* 111:e24122 10.1002/pep2.24122

[B68] Kosiol C. Vinař T. Fonseca R. R. da, Hubisz M. J. Bustamante C. D. (2008). Patterns of positive selection in six mammalian genomes. *PLoS Genet.* 4:e1000144. 10.1371/journal.pgen.1000144 18670650 PMC2483296

[B69] Koziel J. Bryzek D. Sroka A. Maresz K. Glowczyk I. Bielecka E. (2014). Citrullination alters immunomodulatory function of LL-37 essential for prevention of endotoxin-induced sepsis. *J. Immunol.* 192 5363–5372. 10.4049/jimmunol.1303062 24771854 PMC4036085

[B70] Krejner A. Litwiniuk M. Grzela T. (2017). LL-37 but not 25-hydroxy-vitamin D serum level correlates with healing of venous leg ulcers. *Arch. Immunol. Ther. Exp. (Warsz)* 65 455–461. 10.1007/s00005-016-0423-9 27663530 PMC5602047

[B71] Kumar P. Pletzer D. Haney E. F. Rahanjam N. Cheng J. T. J. Yue M. (2019). Aurein-derived antimicrobial peptides formulated with pegylated phospholipid micelles to target methicillin-resistant *Staphylococcus aureus* skin infections. *ACS Infect. Dis.* 5 443–453. 10.1021/acsinfecdis.8b00319 30565465

[B72] Lande R. Ganguly D. Facchinetti V. Frasca L. Conrad C. Gregorio J. (2011). Neutrophils activate plasmacytoid dendritic cells by releasing self-DNA–peptide complexes in systemic lupus erythematosus. *Sci. Transl. Med.* 3 ra19–ra73. 10.1126/scitranslmed.3001180 21389263 PMC3399524

[B73] Lazzaro B. P. Zasloff M. Rolff J. (2020). Antimicrobial peptides: application informed by evolution. *Science* 368:eaau5480. 10.1126/science.aau5480 32355003 PMC8097767

[B74] Lee M. O. Jang H. J. Rengaraj D. Yang S. Y. Han J. Y. Lamont S. J. (2016). Tissue expression and antibacterial activity of host defense peptides in chicken. *BMC Vet. Res.* 12:1–9. 10.1186/s12917-016-0866-6 27737668 PMC5064907

[B75] Levast B. Hogan D. van Kessel J. Strom S. Walker S. Zhu J. (2019). Synthetic cationic peptide IDR-1002 and human cathelicidin LL37 modulate the cell innate response but differentially impact PRRSV replication in vitro. *Front. Vet. Sci.* 6:233. 10.3389/fvets.2019.00233 31355218 PMC6640542

[B76] Libby P. Buring J. E. Badimon L. Hansson G. K. Deanfield J. Bittencourt M. S. (2019). Atherosclerosis. *Nat. Rev. Dis. Primer* 5:56. 10.1038/s41572-019-0106-z 31420554

[B77] Liu C. Qi J. Shan B. Gao R. Gao F. Xie H. (2018). Pretreatment with cathelicidin-BF ameliorates *Pseudomonas aeruginosa* pneumonia in mice by enhancing NETosis and the autophagy of recruited neutrophils and macrophages. *Int. Immunopharmacol.* 65 382–391. 10.1016/j.intimp.2018.10.030 30380513

[B78] Lyle N. H. Pena O. M. Boyd J. H. Hancock R. E. W. (2014). Barriers to the effective treatment of sepsis: antimicrobial agents, sepsis definitions, and host-directed therapies. *Ann. N. Y. Acad. Sci.* 1323 101–114. 10.1111/nyas.12444 24797961

[B79] Mansbach J. M. Hasegawa K. Ajami N. J. Petrosino J. F. Piedra P. A. Tierney C. N. (2017). Serum LL-37 levels associated with severity of bronchiolitis and viral etiology. *Clin. Infect. Dis.* 65 967–975. 10.1093/cid/cix483 28541502 PMC5850627

[B80] Mansour S. C. Pena O. M. Hancock R. E. W. (2014). Host defense peptides: front-line immunomodulators. *Trends Immunol.* 35 443–450. 10.1016/j.it.2014.07.004 25113635

[B81] Marshall J. C. (2014). Why have clinical trials in sepsis failed? *Trends Mol. Med.* 20 195–203. 10.1016/j.molmed.2014.01.007 24581450

[B82] Martin L. Van Meegern A. Doemming S. Schuerholz T. (2015). Antimicrobial peptides in human sepsis. *Front. Immunol.* 6:404. 10.3389/fimmu.2015.00404 26347737 PMC4542572

[B83] Masso-Silva J. A. Diamond G. (2014). Antimicrobial peptides from fish. *Pharm. Basel Switz.* 7 265–310. 10.3390/ph7030265 24594555 PMC3978493

[B84] Meguro S. Tomita M. Katsuki T. Kato K. Oh H. Ainai A. (2014). Plasma antimicrobial peptide LL-37 level is inversely associated with HDL cholesterol level in patients with Type 2 Diabetes Mellitus. *Int. J. Endocrinol.* 2014:703696. 10.1155/2014/703696 24790601 PMC3984791

[B85] Merres J. Höss J. Albrecht L.-J. Kress E. Soehnlein O. Jansen S. (2014). Role of the cathelicidin-related antimicrobial peptide in inflammation and mortality in a mouse model of bacterial meningitis. *J. Innate Immun.* 6 205–218. 10.1159/000353645 23969854 PMC6741491

[B86] Mily A. Rekha R. S. Kamal S. M. M. Akhtar E. Sarker P. Rahim Z. (2013). Oral intake of phenylbutyrate with or without vitamin D3 upregulates the cathelicidin LL-37 in human macrophages: a dose finding study for treatment of tuberculosis. *BMC Pulm. Med.* 13:23. 10.1186/1471-2466-13-23 23590701 PMC3637063

[B87] Mily A. Rekha R. S. Kamal S. M. M. Arifuzzaman A. S. M. Rahim Z. Khan L. (2015). Significant effects of oral phenylbutyrate and vitamin D3 adjunctive therapy in pulmonary tuberculosis: a randomized controlled trial. *PLoS One* 10:e0138340. 10.1371/journal.pone.0138340 26394045 PMC4578887

[B88] Mookherjee N. Anderson M. A. Haagsman H. P. Davidson D. J. (2020). Antimicrobial host defence peptides: functions and clinical potential. *Nat. Rev. Drug Discov.* 19 311–322. 10.1038/s41573-019-0058-8 32107480

[B89] Mookherjee N. Brown K. L. Bowdish D. Doria S. Falsafi R. Hokamp K. (2006). Modulation of the TLR-mediated inflammatory response by the endogenous human host defense peptide LL-37. *J. Immunol.* 176 2455–2464. 10.4049/jimmunol.176.4.2455 16456005

[B90] Mookherjee N. Hamill P. Gardy J. Blimkie D. Falsafi R. Chikatamarla A. (2009). Systems biology evaluation of immune responses induced by human host defence peptide LL-37 in mononuclear cells. *Mol. Biosyst.* 5 483–496. 10.1039/b813787k 19381363

[B91] Mu L. Tang J. Liu H. Shen C. Rong M. Zhang Z. (2014). A potential wound-healing-promoting peptide from salamander skin. *FASEB J.* 28 3919–3929. 10.1096/fj.13-248476 24868009 PMC5395725

[B92] Mu L. Zhou L. Yang J. Zhuang L. Tang J. Liu T. (2017). The first identified cathelicidin from tree frogs possesses anti-inflammatory and partial LPS neutralization activities. *Amino Acids* 49 1571–1585. 10.1007/s00726-017-2449-7 28593346 PMC5561178

[B93] Murakami M. Lopez-Garcia B. Braff M. Dorschner R. A. Gallo R. L. (2004). Postsecretory processing generates multiple cathelicidins for enhanced topical antimicrobial defense. *J. Immunol.* 172 3070–3077. 10.4049/jimmunol.172.5.3070 14978112

[B94] Nell M. J. Tjabringa G. S. Wafelman A. R. Verrijk R. Hiemstra P. S. Drijfhout J. W. (2006). Development of novel LL-37 derived antimicrobial peptides with LPS and LTA neutralizing and antimicrobial activities for therapeutic application. *Peptides* 27 649–660. 10.1016/j.peptides.2005.09.016 16274847

[B95] Ng S. M. S. Teo S. W. Yong Y. E. Ng F. M. Lau Q. Y. Jureen R. (2017). Preliminary investigations into developing all-D Omiganan for treating Mupirocin-resistant MRSA skin infections. *Chem. Biol. Drug Des.* 90 1155–1160. 10.1111/cbdd.13035 28581672

[B96] Nicholls E. F. Madera L. Hancock R. E. W. (2010). Immunomodulators as adjuvants for vaccines and antimicrobial therapy. *Ann. N. Y. Acad. Sci.* 1213 46–61. 10.1111/j.1749-6632.2010.05787.x 20946578

[B97] Nijnik A. Pistolic J. Filewod N. C. J. Hancock R. E. W. (2012). Signaling pathways mediating chemokine induction in keratinocytes by cathelicidin LL-37 and flagellin. *J. Innate Immun.* 4 377–386. 10.1159/000335901 22516952 PMC6741628

[B98] Niyonsaba F. Iwabuchi K. Someya A. Hirata M. Matsuda H. Ogawa H. (2002). A cathelicidin family of human antibacterial peptide LL-37 induces mast cell chemotaxis. *Immunology* 106 20–26. 10.1046/j.1365-2567.2002.01398.x 11972628 PMC1782699

[B99] Nizet V. Ohtake T. Lauth X. Trowbridge J. Rudisill J. Dorschner R. A. (2001). Innate antimicrobial peptide protects the skin from invasive bacterial infection. *Nature* 414 454–457. 10.1038/35106587 11719807

[B100] Osorio D. Rondón-Villarreal P. Torres R. (2015). Peptides: a package for data mining of antimicrobial peptides. *R J* 7:4 10.32614/RJ-2015-001

[B101] Parisi R. Symmons D. P. M. Griffiths C. E. M. Ashcroft D. M. (2013). Global epidemiology of psoriasis: a systematic review of incidence and prevalence. *J. Invest. Dermatol.* 133 377–385. 10.1038/jid.2012.339 23014338

[B102] Park B. W. Ha J. M. Cho E. B. Jin J. K. Park E. J. Park H. R. (2018). A study on vitamin D and cathelicidin status in patients with rosacea: serum level and tissue expression. *Ann. Dermatol.* 30 136–142. 10.5021/ad.2018.30.2.136 29606809 PMC5839883

[B103] Park K. Elias P. M. Oda Y. Mackenzie D. Mauro T. Holleran W. M. (2011). Regulation of cathelicidin antimicrobial peptide expression by an endoplasmic reticulum (ER) stress signaling, vitamin D receptor-independent pathway. *J. Biol. Chem.* 286 34121–34130. 10.1074/jbc.M111.250431 21832078 PMC3190812

[B104] Parker S. J. Watkins P. E. (2001). Experimental models of gram-negative sepsis. *Br. J. Surg.* 88 22–30. 10.1046/j.1365-2168.2001.01632.x 11136305

[B105] Peek F. Nell M. J. Brand R. Jansen-Werkhoven T. Van Hoogdalem E. Frijns J. (2009). “Double-blind placebo-controlled study of the novel peptide drug P60.4Ac in cronic middle ear infection,” in *ICCAC*, (San Francisco, CA, L1–L337.

[B106] Pena O. M. Afacan N. Pistolic J. Chen C. Madera L. Falsafi R. (2013). Synthetic cationic peptide IDR-1018 modulates human macrophage differentiation. *PLoS One* 8:e52449. 10.1371/journal.pone.0052449 23308112 PMC3538731

[B107] Pena O. M. Hancock D. G. Lyle N. H. Linder A. Russell J. A. Xia J. (2014). An endotoxin tolerance signature predicts sepsis and organ dysfunction at initial clinical presentation. *EBioMedicine* 1 64–71. 10.1016/j.ebiom.2014.10.003 25685830 PMC4326653

[B108] Pena O. M. Pistolic J. Raj D. Fjell C. D. Hancock R. E. W. (2011). Endotoxin tolerance represents a distinctive state of alternative polarization (M2) in human mononuclear cells. *J. Immunol.* 186 7243–7254. 10.4049/jimmunol.1001952 21576504

[B109] Polewicz M. Gracia A. Garlapati S. Van Kessel J. Strom S. Halperin S. A. (2013). Novel vaccine formulations against pertussis offer earlier onset of immunity and provide protection in the presence of maternal antibodies. *Vaccine* 31 3148–3155. 10.1016/j.vaccine.2013.05.008 23684829

[B110] Qi R.-H. Chen Y. Guo Z.-L. Zhang F. Fang Z. Huang K. (2019). Identification and characterization of two novel cathelicidins from the frog *Odorrana livida*. *Zool. Res.* 40 94–101. 10.24272/j.issn.2095-8137.2018.062 30127328 PMC6378563

[B111] Qiao X. Yang H. Gao J. Zhang F. Chu P. Yang Y. (2019). Diversity, immunoregulatory action and structure-activity relationship of green sea turtle cathelicidins. *Dev. Compar. Immunol.* 98 189–204. 10.1016/j.dci.2019.05.005 31121185

[B112] Qin X. Zhu G. Huang L. Zhang W. Huang Y. Xi X. (2019). LL-37 and its analog FF/CAP18 attenuate neutrophil migration in sepsis-induced acute lung injury. *J. Cell Biochem.* 120 4863–4871. 10.1002/jcb.27641 30537236

[B113] R Core Team (2020). *R: A Language and Environment for Statistical Computing.* Vienna: R Foundation for Statistical Computing Available online at: https://www.R-project.org/ (accessed May 16, 2020).

[B114] Rachakonda T. D. Schupp C. W. Armstrong A. W. (2014). Psoriasis prevalence among adults in the United States. *J. Am. Acad. Dermatol.* 70 512–516. 10.1016/j.jaad.2013.11.013 24388724

[B115] Ramanathan B. Davis E. G. Ross C. R. Blecha F. (2002). Cathelicidins: microbicidal activity, mechanisms of action, and roles in innate immunity. *Microbes Infect.* 4 361–372. 10.1016/s1286-4579(02)01549-6 11909747

[B116] Rees F. Doherty M. Grainge M. J. Lanyon P. Zhang W. (2017). The worldwide incidence and prevalence of systemic lupus erythematosus: a systematic review of epidemiological studies. *Rheumatology* 56 1945–1961. 10.1093/rheumatology/kex260 28968809

[B117] Rhouma M. Fairbrother J. M. Beaudry F. Letellier A. (2017). Post weaning diarrhea in pigs: risk factors and non-colistin-based control strategies. *Acta Vet. Scand.* 59:31. 10.1186/s13028-017-0299-7 28526080 PMC5437690

[B118] Riedemann N. C. Guo R.-F. Ward P. A. (2003). The enigma of sepsis. *J. Clin. Invest.* 112 460–467. 10.1172/JCI200319523 12925683 PMC171398

[B119] Riool M. De Breij A. Drijfhout J. W. Nibbering P. H. Zaat S. A. J. (2017). Antimicrobial peptides in biomedical device manufacturing. *Front. Chem.* 5:63. 10.3389/fchem.2017.00063 28971093 PMC5609632

[B120] Rittirsch D. Flierl M. A. Ward P. A. (2008). Harmful molecular mechanisms in sepsis. *Nat. Rev. Immunol.* 8 776–787. 10.1038/nri2402 18802444 PMC2786961

[B121] Rivas-Santiago B. Rivas Santiago C. E. Castañeda-Delgado J. E. León-Contreras J. C. Hancock R. E. W. Hernandez-Pando R. (2013). Activity of LL-37, CRAMP and antimicrobial peptide-derived compounds E2, E6 and CP26 against *Mycobacterium tuberculosis*. *Int. J. Antimicrob. Agents* 41 143–148. 10.1016/j.ijantimicag.2012.09.015 23141114

[B122] Rivas-Santiago B. Trujillo V. Montoya-Rosales A. Gonzalez-Curiel I. Castañeda-Delgado J. Cardenas A. (2012). Expression of antimicrobial peptides in diabetic foot ulcer. *J. Dermatol. Sci.* 65 19–26. 10.1016/j.jdermsci.2011.09.013 22047630

[B123] Ron-Doitch S. Sawodny B. Kühbacher A. David M. M. N. Samanta A. Phopase J. (2016). Reduced cytotoxicity and enhanced bioactivity of cationic antimicrobial peptides liposomes in cell cultures and 3D epidermis model against HSV. *J. Controlled Release* 229 163–171. 10.1016/j.jconrel.2016.03.025 27012977

[B124] Rubinchik E. Dugourd D. Algara T. Pasetka C. Friedland H. D. (2009). Antimicrobial and antifungal activities of a novel cationic antimicrobial peptide, omiganan, in experimental skin colonisation models. *Int. J. Antimicrob. Agents* 34 457–461. 10.1016/j.ijantimicag.2009.05.003 19524411

[B125] Rudd K. E. Johnson S. C. Agesa K. M. Shackelford K. A. Tsoi D. Kievlan D. R. (2020). Global, regional, and national sepsis incidence and mortality, 1990–2017: analysis for the Global Burden of Disease Study. *Lancet* 395 200–211. 10.1016/S0140-6736(19)32989-7 31954465 PMC6970225

[B126] Savonius O. Helve O. Roine I. Andersson S. Saukkoriipi A. González Mata A. (2018). Cerebrospinal fluid cathelicidin correlates with the bacterial load and outcomes in childhood bacterial meningitis. *Pediatr. Infect. Dis. J.* 37 182–185. 10.1097/INF.0000000000001744 28827496

[B127] Schlosser E. Mueller M. Fischer S. Basta S. Busch D. H. Gander B. (2008). TLR ligands and antigen need to be coencapsulated into the same biodegradable microsphere for the generation of potent cytotoxic T lymphocyte responses. *Vaccine* 26 1626–1637. 10.1016/j.vaccine.2008.01.030 18295941

[B128] Schön M. P. (2019). Adaptive and innate immunity in psoriasis and other inflammatory disorders. *Front. Immunol.* 10:1764. 10.3389/fimmu.2019.01764 31402919 PMC6676248

[B129] Schrumpf J. A. Sterkenburg M. A. J. A. V. Verhoosel R. M. Zuyderduyn S. Hiemstra P. S. (2012). Interleukin 13 exposure enhances vitamin D-mediated expression of the human cathelicidin antimicrobial peptide 18/LL-37 in bronchial epithelial cells. *Infect. Immun.* 80 4485–4494. 10.1128/IAI.06224-11 23045480 PMC3497402

[B130] Scott M. G. Davidson D. J. Gold M. R. Bowdish D. Hancock R. E. W. (2002). The human antimicrobial peptide LL-37 is a multifunctional modulator of innate immune responses. *J. Immunol.* 169 3883–3891. 10.4049/jimmunol.169.7.3883 12244186

[B131] Shi N. Cai S. Gao J. Qiao X. Yang H. Wang Y. (2019). Roles of polymorphic cathelicidins in innate immunity of soft-shell turtle, *Pelodiscus sinensis*. *Dev. Compar. Immunol.* 92 179–192. 10.1016/j.dci.2018.11.010 30452933

[B132] Shi Y. Li C. Wang M. Chen Z. Luo Y. Xia X. (2020). Cathelicidin-DM is an antimicrobial peptide from *Duttaphrynus melanostictus* and has wound-healing therapeutic potential. *ACS Omega* 5 9301–9310. 10.1021/acsomega.0c00189 32363280 PMC7191562

[B133] Singh D. Vaughan R. Kao C. C. (2014). LL-37 peptide enhancement of signal transduction by toll-like receptor 3 is regulated by pH identification of a peptide antagonist of LL-37. *J. Biol. Chem.* 289 27614–27624. 10.1074/jbc.M114.582973 25092290 PMC4183800

[B134] Sinha M. Kaushik S. Kaur P. Sharma S. Singh T. P. (2013). Antimicrobial lactoferrin peptides: the hidden players in the protective function of a multifunctional protein. *Int. J. Pept.* 2013:390230. 10.1155/2013/390230 23554820 PMC3608178

[B135] Sørensen O. E. Follin P. Johnsen A. H. Calafat J. Tjabringa G. S. Hiemstra P. S. (2001). Human cathelicidin, hCAP-18, is processed to the antimicrobial peptide LL-37 by extracellular cleavage with proteinase 3. *Blood* 97 3951–3959. 10.1182/blood.V97.12.3951 11389039

[B136] Sun C.-L. Zhang F.-Z. Li P. Bi L.-Q. (2011). LL-37 expression in the skin in systemic lupus erythematosus. *Lupus* 20 904–911. 10.1177/0961203311398515 21562016

[B137] Suzuki K. Murakami T. Kuwahara-Arai K. Tamura H. Hiramatsu K. Nagaoka I. (2011). Human anti-microbial cathelicidin peptide LL-37 suppresses the LPS-induced apoptosis of endothelial cells. *Int. Immunol.* 23 185–193. 10.1093/intimm/dxq471 21393634

[B138] Travis S. M. Anderson N. N. Forsyth W. R. Espiritu C. Conway B. D. Greenberg E. P. (2000). Bactericidal activity of mammalian cathelicidin-derived peptides. *Infect. Immun.* 68 2748–2755. 10.1128/IAI.68.5.2748-2755.2000 10768969 PMC97484

[B139] Tripathi S. Wang G. White M. Qi L. Taubenberger J. Hartshorn K. L. (2015). Antiviral activity of the human cathelicidin, LL-37, and derived peptides on seasonal and pandemic influenza A viruses. *PLoS One* 10:e0124706. 10.1371/journal.pone.0124706 25909853 PMC4409069

[B140] Uzzell T. Stolzenberg, Shinnar A. E. Zasloff M. (2003). Hagfish intestinal antimicrobial peptides are ancient cathelicidins. *Peptides* 24 1655–1667. 10.1016/j.peptides.2003.08.024 15019197

[B141] Van der Poll T. van de Veerdonk F. L. Scicluna B. P. Netea M. G. (2017). The immunopathology of sepsis and potential therapeutic targets. *Nat. Rev. Immunol.* 17 407–420. 10.1038/nri.2017.36 28436424

[B142] Van Dijk A. Hedegaard C. J. Haagsman H. P. Heegaard P. M. H. (2018). The potential for immunoglobulins and host defense peptides (HDPs) to reduce the use of antibiotics in animal production. *Vet. Res.* 49 1–16. 10.1186/s13567-018-0558-2 30060758 PMC6066942

[B143] Van Harten R. M. Van Woudenbergh E. Van Dijk A. Haagsman H. P. (2018). Cathelicidins: immunomodulatory antimicrobials. *Vaccines* 6:63. 10.3390/vaccines6030063 30223448 PMC6161271

[B144] Venet F. Monneret G. (2018). Advances in the understanding and treatment of sepsis-induced immunosuppression. *Nat. Rev. Nephrol.* 14 121–137. 10.1038/nrneph.2017.165 29225343

[B145] Verjans E.-T. Zels S. Luyten W. Landuyt B. Schoofs L. (2016). Molecular mechanisms of LL-37-induced receptor activation: an overview. *Peptides* 85 16–26. 10.1016/j.peptides.2016.09.002 27609777

[B146] Wang T.-T. Nestel F. P. Bourdeau V. Nagai Y. Wang Q. Liao J. (2004). Cutting edge: 1,25-dihydroxyvitamin D 3 is a direct inducer of antimicrobial peptide gene expression. *J. Immunol.* 173 2909–2912. 10.4049/jimmunol.173.5.2909 15322146

[B147] Wu J. Yang J. Wang X. Wei L. Mi K. Shen Y. (2018). A frog cathelicidin peptide effectively promotes cutaneous wound healing in mice. *Biochem. J.* 475 2785–2799. 10.1042/BCJ20180286 30045878 PMC6134359

[B148] Xiao Y. Cai Y. Bommineni Y. R. Fernando S. C. Prakash O. Gilliland S. E. (2006). Identification and functional characterization of three chicken cathelicidins with potent antimicrobial activity. *J. Biol. Chem.* 281 2858–2867. 10.1074/jbc.M507180200 16326712

[B149] Xu X. Lai R. (2015). The chemistry and biological activities of peptides from amphibian skin secretions. *Chem. Rev.* 115 1760–1846. 10.1021/cr4006704 25594509

[B150] Yamasaki K. Di Nardo A. Bardan A. Murakami M. Ohtake T. Coda A. (2007). Increased serine protease activity and cathelicidin promotes skin inflammation in rosacea. *Nat. Med.* 13 975–980. 10.1038/nm1616 17676051

[B151] Yamasaki K. Schauber J. Coda A. Lin H. Dorschner R. A. Schechter N. M. (2006). Kallikrein-mediated proteolysis regulates the antimicrobial effects of cathelicidins in skin. *FASEB J.* 20 2068–2080. 10.1096/fj.06-6075com 17012259

[B152] Yang D. Chen Q. Schmidt A. P. Anderson G. M. Wang J. M. Wooters J. (2000). LL-37, the neutrophil granule–and epithelial cell–derived cathelicidin, utilizes formyl peptide receptor–like 1 (Fprl1) as a receptor to chemoattract human peripheral blood neutrophils, monocytes, and T Cells. *J. Exp. Med.* 192 1069–1074. 10.1084/jem.192.7.1069 11015447 PMC2193321

[B153] Yi H. Zhang L. Gan Z. Xiong H. Yu C. Du H. (2016). High therapeutic efficacy of Cathelicidin-WA against postweaning diarrhea via inhibiting inflammation and enhancing epithelial barrier in the intestine. *Sci. Rep.* 6:25679. 10.1038/srep25679 27181680 PMC4867772

[B154] Yu K. Lo J. C. Y. Mei Y. Haney E. F. Siren E. Kalathottukaren M. T. (2015). Toward infection-resistant surfaces: achieving high antimicrobial peptide potency by modulating the functionality of polymer brush and peptide. *ACS Appl. Mater. Interfaces* 7 28591–28605. 10.1021/acsami.5b10074 26641308

[B155] Yu Y. Zhang Y. Zhang Y. Lai Y. Chen W. Xiao Z. (2017). LL-37-induced human mast cell activation through G protein-coupled receptor MrgX2. *Int. Immunopharmacol.* 49 6–12. 10.1016/j.intimp.2017.05.016 28549244

[B156] Zanetti M. (2005). The role of cathelicidins in the innate host defenses of mammals. *Curr. Issues Mol. Biol.* 7 179–196. 10.21775/cimb.007.179 16053249

[B157] Zapotoczna M. Forde É Hogan S. Humphreys H. O’gara J. P. Fitzgerald-Hughes D. (2017). Eradication of *Staphylococcus aureus* biofilm infections using synthetic antimicrobial peptides. *J. Infect. Dis.* 215 975–983. 10.1093/infdis/jix062 28453851

[B158] Zhang Q. Tang J. Ran R. Liu Y. Zhang Z. Gao H. (2016). Development of an anti-microbial peptide-mediated liposomal delivery system: a novel approach towards pH-responsive anti-microbial peptides. *Drug Deliv.* 23 1163–1170. 10.3109/10717544.2014.1003665 25693639

[B159] Zhang Z. Meng P. Han Y. Shen C. Li B. Hakim M. A. (2015). Mitochondrial DNA-LL-37 complex promotes atherosclerosis by escaping from autophagic recognition. *Immunity* 43 1137–1147. 10.1016/j.immuni.2015.10.018 26680206

[B160] Zhu S. Gao B. (2017). Positive selection in cathelicidin host defense peptides: adaptation to exogenous pathogens or endogenous receptors? *Heredity* 118 453–465. 10.1038/hdy.2016.117 27925615 PMC5564380

