# Supplementary material for: Cathelicidin Host Defense Peptides and Inflammatory Signaling: Striking a Balance
Source: Front Microbiol. 2020 Aug 27;11:1902. doi: 10.3389/fmicb.2020.01902 (PMC7481365; doi:10.3389/fmicb.2020.01902)
Supplement: Supplementary file 1 [file Table_1.docx]

**Supplementary Information**

**­Cathelicidin Host Defense Peptides and Inflammatory Signaling: Striking a Balance**

Morgan A Alford*^1^, Beverlie Baquir*^1^, Felix L Santana^1,2^, Evan F Haney^1^ and Robert EW Hancock^1#^

* These authors contributed equally to this work

^1^Centre for Microbial Diseases and Immunity Research, University of British Columbia, 2259 Lower Mall Research Station, Vancouver, BC, V6T1Z4

^2^Departamento de Medicina Molecular y Bioprocesos, Instituto de Biotecnología, Universidad Nacional Autónoma de México, Avenida Universidad 2001, Colonia Chamilpa, Cuernavaca, Morelos, 62210, Mexico.

# Corresponding author: bob@hancocklab.com

**Table of Contents:**

| **Pages** | **Description** |
| --- | --- |
| 2-9 | Table S1 - Physicochemical properties and structural classification of representative vertebrate cathelicidin peptides |
| 10 | Figure S1 - Distribution of select amino acid residues among representative vertebrate cathelicidins. |
| 11-15 | Table S2 - Percent proportion of select amino acids in representative vertebrate cathelicidins. |
| 16-18 | Supplementary References |

**Table S1** - **Physicochemical properties and structural classification of representative vertebrate cathelicidin peptides**. Cysteine residues forming disulfide bridges are highlighted in bold. In the case of Protegrin-1, residues involved in each disulfide bridge are numbered. Physico-chemical properties were computed using the *Peptides* package v2.4.2 (1) in R v4.0.0 (2). Net charge was calculated using Bjellqvist’s pK scale implemented in the *Peptides* package.

| **Peptide Name** | **Sequence** | **Species**  **(common name)** | **Length** | **Net charge** | **Hydro-phobic residues (%)** | **Structural class** | **APD #, (Ref.)** |
| --- | --- | --- | --- | --- | --- | --- | --- |
| **Mammals** | | | | | | | |
| Dodecapeptide | RL**C**RIVVIRV**C**R | Cattle | 12 | 3.7 | 67 | Cyclic, disulfide bridged | 00008 |
| Indolicidin | ILPWKWPWWPWRR-NH_2_ | Cattle | 13 | 3.8 | 54 | Trp-rich# | 00150, (3) |
| buCATHL4A | GLPWILLRWLFFR-NH_2_ | Water Buffalo | 13 | 2.8 | 69 | α-helical (4) | 02638, (3) |
| Protegrin-1 | RGGRL**C_1_**Y**C_2_**RRRF**C_2_**V**C_1_**VGR-NH_2_ | Pig | 18 | 6.7 | 50 | β-structured, disulfide bridged | 00195, (3) |
| PMAP-23 | RIIDLLWRVRRPQKPKFVTVWVR | Pig | 23 | 5.8 | 48 | α-helical | 00369 |
| BMAP-27 | GRFKRFRKKFKKLFKKLSPVIPLLHL-NH_2_ | Cattle | 26 | 10.8 | 42 | α-helical | 00366, (3) |
| eCATH-2 | KRRHWFPLSFQEFLEQLRRFRDQLPFP | Horse | 27 | 2.9 | 37 | α-helical | 00687 |
| SMAP-29 | RGLRRLGRKIAHGVKKYGPTVLRIIRIAG-NH_2_ | Sheep | 28 | 9.8 | 43 | α-helical | 00155, (3) |
| mCRAMP | GLLRKGGEKIGEKLKKIGQKIKNFFQKLVPQPEQ | Mouse | 34 | 5.8 | 29 | α-helical | 00281 |
| rCRAMP | GLVRKGGEKFGEKLRKIGQKIKEFFQKLALEIEQ | Rat | 34 | 3.8 | 35 | Predicted  α-helical (5) | 00498 |
| PMAP-36 | GRFRRLRKKTRKRLKKIGKVLKWIPPIVGSIPLGC-NH_2_ | Pig | 35 | 13.7 | 37 | α-helical | (3) |
| LL-37 | LLGDFFRKSKEKIGKEFKRIVQRIKDFLRNLVPRTES | Human | 37 | 5.8 | 35 | α-helical | 00310 |
| CAP18-FV | GLRKRLRKFRNKIKEKLKKIGQKIQGFVPKLAPRTDY | Rabbit | 37 | 11.8 | 32 | Predicted  α-helical | (5) |
| PMAP-37 | GLLSRLRDFLSDRGRRLGEKIERIGQKIKDLSEFFQS | Pig | 37 | 2.8 | 32 | α-helical | 00371, (3) |
| ttLL-37 | RLGDFLRRGGEKTGKKIERIGQRIKDFFGIFQPSKQS | Common bottlenose dolphin | 37 | 5.8 | 27 | Predicted  α-helical | (5) |
| eCATH-3 | KRFHSVGSLIQRHQQMIRDKSEATRHGIRIITRPKLLLAS | Horse | 40 | 7.0 | 35 | α-helical | 00688 |
| Bac7 | RRIRPRPPRLPRPRPRPLPFPRPGPRPIPRPLPFPRPGPRPIPRPLPFPRPGPRPIPRPL | Cattle | 60 | 16.8 | 20 | Pro-rich | 00010 |
| Prophenin-1 | AFPPPNVPGPRFPPPNFPGPRFPPPNFPGPRFPPPNFPGPRFPPPNFPGPPFPPPIFPGPWFPPPPPFRPPPFGPPRFP-NH_2_ | Pig | 79 | 6.8 | 24 | Pro-rich | 00689, (3) |
| **Birds** | | | | | | | |
| dCATH | KRFWQLVPLAIKIYRAWKRR | American Pekin Duck | 20 | 6.8 | 55 | α-helical | 02629 |
| CATHL3-CUCCA | RVKRLWPLLTVAFGIFKSIKLKT | Common Cuckoo | 23 | 5.8 | 52 | ND | (6) |
| chCATH-1 | RVKRVWPLVIRTVIAGYNLYRAIKKK | Chicken | 26 | 7.8 | 54 | α-helical | 00557 |
| chCATH-2 | RFGRFLRKIRRFRPKVTITIQGSARF | Chicken | 26 | 8.8 | 38 | α-helical | 00548 |
| Pc-CATH1 | RIKRFWPVVIRTVVAGYNLYRAIKKK | Ring-necked Pheasant | 26 | 7.8 | 54 | Predicted  α-helical (7) | 01643 |
| CATHL3-AMAVI | RVKRFWPLLVTAIRTVAAGVGIFKSFKG | Puerto Rican Parrot | 28 | 5.8 | 54 | ND | (6) |
| chCATH-3 | RVKRFWPLVPVAINTVAAGINLYKAIRRK | Chicken | 29 | 6.8 | 55 | α-helical | 00613 |
| cc-CATH3 | RVRRFWPLVPVAINTVAAGINLYKAIRRK | Common Quail | 29 | 6.8 | 55 | Predicted  α-helical (7) | 01750 |
| CATHL3-BUCRH | QVKRFWLLVPVAIKTVATSINHFKPKERK | Rhinoceros Hornbil | 29 | 5.8 | 45 | ND | (6) |
| cc-CATH2 | LVQRGRFGRFLKKVRRFIPKVIIAAQIGSRFG | Common Quail | 32 | 8.8 | 47 | Predicted  α-helical (7) | 01749 |
| CATHL2-COLVI | LVQRGRFRRFLGKIRRYLPKFVIAVQGGTRFG | Northern Bobwhite | 32 | 8.8 | 44 | ND | (6) |
| CATHL2-ACACH | LIQRGRFGRFLSKIRRFRPKINFNVHTRVSVGLG | Rifleman | 34 | 8.8 | 38 | ND | (6) |
| Cl-CATH2 | LIQRGRFGRFLGRIRRFRPRINFDIRARGSIRLG | Rock pigeon | 34 | 9.8 | 38 | α-helical | 02626 |
| CATHL2-PSEHU | LIQRGRFGRFLGKIRHFRPRVKFDIRLKGSVGLG | Tibetan ground-tit | 34 | 8.8 | 38 | ND | (6) |
| CATHB1-CAPCA | FGRIRRSKVRGLLTKIKDGLRSFFQCSKIWIRDKLNLN | Chuck-will’s- widow | 38 | 8.7 | 39 | ND | (6) |
| chCATH-B1 | PIRNWWIRIWEWLNGIRKRLRQRSPFYVRGHLNVTSTPQP | Chicken | 40 | 6.8 | 38 | ND | (8) |
| CATHB1-CHAVO | LGRIQTSRLKDFFAKIKERFRGFFQCGKIWIRDKLNLTKPQP | Killdeer | 42 | 7.7 | 38 | ND | (6) |
| CATHB1-COLLI | VGRIRPSPLRDLLARIRDQLRNIIPCGNIWIRDKLNLQPPKP | Rock pigeon | 42 | 5.8 | 38 | ND | (6) |
| **Reptiles** | | | | | | | |
| As-CATH5 | TRRKFWKKVLNGALKIAPFLLG | Chinese Alligator | 22 | 5.8 | 50 | α-helical | 02899 |
| As-CATH6 | TRWLWLLRGGLKAAGWGIRAHLNRNQ | Chinese Alligator | 26 | 4.8 | 46 | α-helical | 02900 |
| Cathelicidin-BF | KFFRKLKKSVKKRAKEFFKKPRVIGVSIPF | Banded Krait | 30 | 10.8 | 40 | α-helical | 01239 |
| Hc-CATH | KFFKRLLKSVRRAVKKFRKKPRLIGLSTLL | Annulated Sea Snake | 30 | 11.8 | 43 | α-helical | 02569 |
| SA-CATH | KFFKKLKKSVKKHVKKFFKKPKVIGVSIPF | Red-bellied annulate keelback | 30 | 11.8 | 40 | Predicted  α-helical | (9) |
| CATHPb1 | KRFKKFFRKIKKGFRKIFKKTKIFIGGTIPI | Burmese Python | 31 | 12.8 | 39 | α-helical | 02964 |
| As-CATH3 | PAKPKPRPGKLSSFTLHLAPGSDGKPRCHYP | Chinese Alligator | 31 | 4.9 | 26 | Non regular structured | (10)(6) |
| Ps-CATH4 | TRGRWGRFKRRAGRFIRRNRWQIISTGLKLIG | Chinese Softshell Turtle | 32 | 10.8 | 34 | α-helical | 03050 |
| Cm-CATH2 | RRSRFGRFFKKVRKQLGRVLRHSRITVGGRMRF | Green Sea Turtle | 33 | 12.8 | 33 | Predicted α‑helical, β‑structured (11) | 03079 |
| Cm-CATH3 | TRGRWKRFWRGAGRFFRRHKEKIIRAAVDIVLS | Green Sea Turtle | 33 | 8.8 | 42 | Predicted α‑helical (11) | 03080 |
| NA-CATH | KRFKKFFKKLKNSVKKRAKKFFKKPKVIGVTFPF | Chinese Cobra | 34 | 14.7 | 38 | α-helical | 00897 |
| Ps-CATH6 | KKPSKKPKPQAMTFPKVTVEYFPASFSTAALTVPED | Chinese Softshell Turtle | 36 | 2.8 | 36 | α-helical | 03051 |
| KP36 | KPKPKPGKDERGRPGSGSWIGKGTPFSFPITKKPVG | Siamese Crocodile | 36 | 6.8 | 17 | Predicted α‑helical, β‑structured | (12) |
| As-CATH2 | RRSGWWNGHKRRRGSGTRRGRFSHIAHGGRKGHERIA | Chinese Alligator | 37 | 11.1 | 19 | Non regular structured | (10) |
| As-CATH4 | RRGLFKKLRRKIKKGFKKIFKRLPPVGVGVSIPLAGRR | Chinese Alligator | 38 | 14.8 | 37 | α-helical | 02898 |
| Cm-CATH1 | RRSIFRKLRRKIKKGLKKGIQHLLAGGRQGLPQGGRPGMI | Green Sea Turtle | 40 | 12.8 | 30 | Predicted α‑helical (11) | 03078 |
| Cm-CATH4 | MAFPFSTQRINPEIEEGNASLADLPVTHAGSLPGIKAQVRTALGIALLLVA | Green Sea Turtle | 51 | -1.1 | 49 | Predicted α‑helical (11) | 03081 |
| As-CATH1 | RRSGWWNGHKRRRGSGSRHGQYSSTKYGGRKRPRKRPGSGSWLSHDTPHVAPIAKGHVG | Chinese Alligator | 59 | 14.2 | 19 | Non regular structured | (10) |
| **Amphibians** | | | | | | | |
| Tylotoin | KCVRQNNKRVCK | Himalayan Newt | 12 | 4.7 | 33 | ND | (13) |
| Cathelicidin-NV | ARGKKE**C**KDDR**C**RLLMKRGSFSYV | Plateau Frog | 24 | 4.7 | 38 | ND, disulfide bridged | (14) |
| Cathelicidin-OA1 | IGRDPTWSHLAAS**C**LK**C**IFDDLPKTHN | Golden Cross Band Frog | 27 | -0.1 | 41 | ND, disulfide bridged | (15) |
| Cathelicidin-RC1 | KK**C**KFF**C**KVKKKIKSIGFQIPIVSIPFK | American Bullfrog | 28 | 8.7 | 46 | α-helical, disulfide bridged* | 02456 |
| Cathelicidin-PY | RK**C**NFL**C**KLKEKLRTVITSHIDKVLRPQG | Yunan Spiny Frog | 29 | 5.8 | 38 | α-helical, disulfide bridged* | 02202 |
| Lf-CATH1 | PP**C**RGIF**C**RRVGSSSAIARPGKTLSTFITV | Fragile Wart Frog | 30 | 4.7 | 40 | Predicted α‑helical, disulfide bridged* (16) | 02306 |
| Lf-CATH2 | GK**C**NVL**C**QLKQKLRSIGSGSHIGSVVLPRG | Fragile Wart Frog | 30 | 4.8 | 37 | Predicted α‑helical, disulfide bridged* (16) | 02307 |
| Cathelicidin-PP | ASENGK**C**NLL**C**LVKKKLRAVGNVIKTVVGKIA | Tree Frog | 32 | 5.7 | 50 | β-structured, disulfide bridged* | 02901 |
| Cathelicidin-RC2 | KK**C**GFF**C**KLKNKLKSTGSRSNIAAGTHGGTFRV | American Bullfrog | 33 | 7.8 | 33 | Predicted α‑helical and β‑structured, disulfide bridged* (17) | 02457 |
| OL-CATH2 | RK**C**NFL**C**KVKNKLKSVGSKSLIGSATHHGIYRV | Green Mountain Frog | 33 | 7.9 | 39 | α-helical, , disulfide bridged* (18) | 03012 |
| OL-CATH1 | KK**C**KGYR**C**RPVGFSSPISRRINDSENIYLPFGV | Green Mountain Frog | 33 | 4.7 | 36 | Non regular structured, disulfide bridged* | (18) |
| Cathelicidin-Bg | RPCRGRSCSPWLRGAYTLIGRPAKNQNRPKYMWV | Asiatic Toad | 34 | 7.7 | 38 | ND | (19) |
| AdCath | RPKKVQGRKAEKDNGDGTTAANASGKKKSSNVFK | Chinese Giant Salamander | 34 | 6.8 | 21 | Predicted α‑helical (20) | 02897 |
| BG-CATH37 | SSRRP**C**RGRS**C**GPRLRGGYTLIGRPVKNQNRPKYMWV | Asiatic Toad | 37 | 9.7 | 30 | Predicted β‑structured, disulfide bridged* (21) | 02580 |
| Cathelicidin-DM | SSRRKPCKGWLCKLKLRGGYTLIGSATNLNRPTYVRA | Asian common toad | 37 | 8.7 | 38 | Predicted β‑structured | (22) |
| Cathelicidin-AL | RRSRRGRGGGRRGGSGGRGGRGGGGRSGAGSSIAGVGSRGGGGGRHYA | Rufous-spotted Torrent Frog | 48 | 11.8 | 12 | Gly-rich^#^ | 01898 |
| **Fish** | | | | | | | |
| HFIAP-3 | GWFKKAWRKVKNAGRRVLKGVGIHYGVGLI | Atlantic Hagfish | 30 | 7.8 | 47 | ND | 00692 |
| HFIAP-1 | GFFKKAWRKVKHAGRRVLDTAKGVGRHYVNNWLNRYR | Atlantic Hagfish | 37 | 9.9 | 41 | ND | 00691 |
| AdCATH | RVKRGGNGSRGREGGRPGSRSSTGKPSKSLNFVERLLM | Yangtze sturgeon | 38 | 7.8 | 18 | ND | (23) |
| CATH-THYTH | RRSKSSSNGGRKGSKGGSKGRPGSGSSIAGASGVNHGGTRTA | Grayling | 42 | 8.8 | 12 | Gly-rich^#^ | (24) |
| JeCATH | RRSKAGKGSGGNKGNKGSGGNKGNKGSRPGGGSSIAGRDKGDSGTRTA | Japanese Eel | 48 | 9.8 | 8 | Gly-rich^#^ | (25) |
| CATH-ONCTS | QKIRTRRGKDSGGSRGSKMWGWRGRPGSRSRPGVGSGIAGASGGNHVGTLTA | Chinook Salmon | 52 | 9.8 | 21 | Gly-rich^#^ | (24) |
| AsCATH-2 | RRGKPSGGSRGSKMGSKDSKGGWRGRPGSGSRPGFGSSIAGASGRDQGGTRNA | Atlantic Salmon | 53 | 9.8 | 13 | Gly-rich^#^ | (25) |
| AcCATH | RRGKASGGSSDSNMGRRDSKGGRRGRPGSGSRPGFGSSIAGASGVNHGGTRTA | Arctic Char | 53 | 8.9 | 15 | Gly-rich^#^ | (25) |
| CATH-BRALE | RRSKARGGSRGSKMGRKDSKGGSRGRPGSGSRPGGGSSIAGASRGDRGGTRNA | Lenok Trout | 53 | 12.8 | 11 | Predicted α‑helical, β‑structured (20), Gly‑rich^#^ | 02175 |
| CodCATH-2 | RRSRSGRGSGKGGRGGSRESSGSRGSRGSKGSRGGLGSTIGRNLKKRRTCPVRPL | Atlantic Cod | 55 | 15.7 | 11 | Gly-rich^#^ | (25) |
| BtrCATH | RRSQARK**C**SRGNGGGIR**C**PGGGIRLGGGSLIGRPKGGSPPGGGSFTAGFIRDQRDGNRFA | River Trout | 60 | 9.7 | 23 | Gly-rich^#^, disulfide bridged* | (25) |
| aCATH | RMRRSKSGKGSGGSKGSGSKGSKGSKGSGSKGSGSKGGSRPGGGSSIAGGGSKGKGGTQTA | Ayu | 61 | 13.8 | 7 | Gly-rich^#^,  Ser-rich^#^ | 01759 |
| AsCATH-1 | RRSQARK**C**SRGNGGKIGSIR**C**RGGGTRLGGGSLIGRLRVALLLGVAPFLLDLSQINVMEIAFA | Atlantic Salmon | 63 | 8.7 | 43 | Gly-rich^#^, disulfide bridged* | (25) |
| rtCATH-2a | RRGKDSGGPKMGRKDSKGCWRGRPGSGSRPGFGSGIAGASGVNHVGTLPASNSTTHPLDNCKISPQ | Rainbow Trout | 66 | 7.9 | 21 | β‑structured (26) | 02539 |
| rtCATH-1 | RI**C**SRDKN**C**VSRPGVGSIIGRPGGGSLIGRPGGGSVIGRPGGGSPPGGGSFNDEFIRDHSDGNRFA | Rainbow trout | 66 | 3.8 | 24 | Gly-rich^#^, disulfide bridged* (27) | 00693 |
| CodCATH-1 | RRSRSGRGSGKGGRGGSRGSSGSRGSKGPSGSRGSSGSRGSKGSRGGRSGRGSTIAGNGNRNNGGTRTA | Atlantic Cod | 69 | 16.8 | 4 | Gly-rich^#^,  Ser-rich^#^ | (25) |
| CodCATH-3 | RRSRSGRGSGKGGRGGSRGSSGSRGSKGPSGSRGSSGSRGSKGSSGSRGSKGSRGGRSGRGSTIAGNGNRNNGGTR | Atlantic Cod | 76 | 18.8 | 3 | Gly-rich^#^,  Ser-rich^#^ | (25) |
| CATH1-SALFO | RRSKARI**C**SRGKD**C**KFRSNGRHGSGSRLGGGSLIGRPGGGSRPGSSSVIGRPGGGSRLGSGSLIGRPGGGSRTGVAP | Brook Trout | 77 | 14.8 | 19 | Gly-rich^#^, disulfide bridged* | (24) |

Length = number of amino acids; ND = not determined; Trp = tryptophan; Pro = proline; Gly = glycine; * = disulfide bridge not experimentally confirmed; # = 25% or more of total residues; -NH_2_ denotes C-terminal amidation according to Zanetti *et al*. (3); APD: Antimicrobial Peptide Data Base^1^ (28).

^1^ <http://aps.unmc.edu/AP/main.php>

**
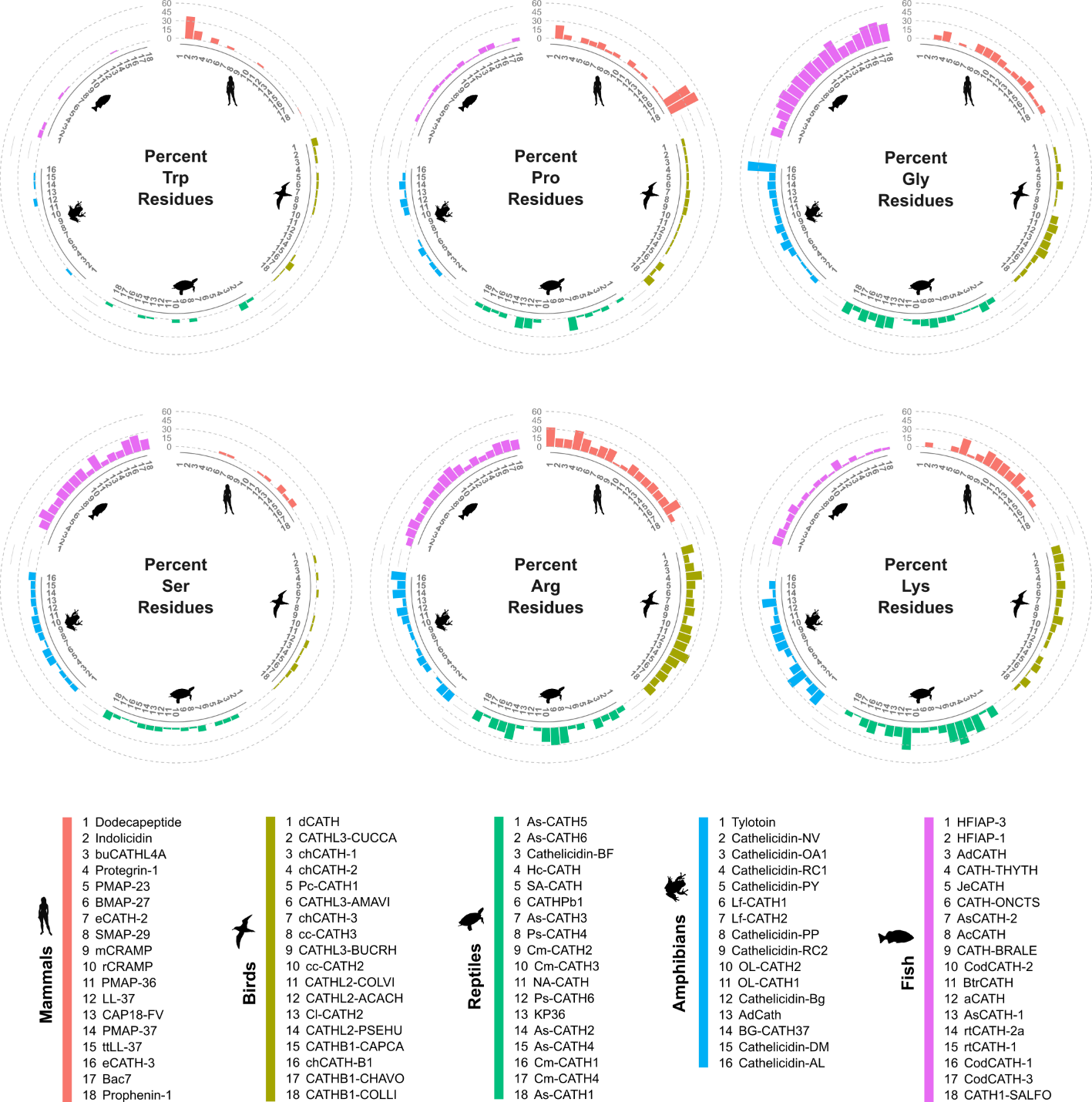
Figure S1 - Distribution of select amino acid residues among representative vertebrate cathelicidins.** Circular bar plots show the proportion of tryptophan (Trp), proline (Pro), glycine (Gly), serine (Ser), arginine (Arg) and lysine (Lys) residues among representative vertebrate cathelicidin peptides listed in Table S1. The order of sequences in each group is sorted by peptide length. Proportions were computed in R v4.0.0 (2) using the *Peptides* package v2.4.2 (1). Animal silhouettes were created by NASA (mammals: human), Juan Carlos Jerí (birds: shearwater), uncredited (reptiles: turtle), Will Booker (amphibians: tree frog) and Felix Vaux (fish), and downloaded from <http://phylopic.org/>.

**Table S2 - Percent proportion of select amino acids in representative vertebrate cathelicidins.** Percent values equal to or greater than 25 are highlighted in bold.

| **Peptide Name** | **% Trp** | **% Gly** | **% Pro** | **% Phe** | **% Arg** | **% Lys** | **% His** | **% Ile** | **% Leu** | **% Val** | **% Ala** | **% Ser** |
| --- | --- | --- | --- | --- | --- | --- | --- | --- | --- | --- | --- | --- |
| **Mammals** | | | | | | | | | | | | |
| Dodecapeptide | 0 | 0 | 0 | 0 | **33** | 0 | 0 | 17 | 8 | **25** | 0 | 0 |
| Indolicidin | **38** | 0 | 23 | 0 | 15 | 8 | 0 | 8 | 8 | 0 | 0 | 0 |
| buCATHL4A | 15 | 8 | 8 | 15 | 15 | 0 | 0 | 8 | **31** | 0 | 0 | 0 |
| Protegrin-1 | 0 | 17 | 0 | 6 | **33** | 0 | 0 | 0 | 6 | 11 | 0 | 0 |
| PMAP-23 | 9 | 0 | 9 | 4 | 22 | 9 | 0 | 9 | 9 | 17 | 0 | 0 |
| BMAP-27 | 0 | 4 | 8 | 15 | 12 | **27** | 4 | 4 | 19 | 4 | 0 | 4 |
| eCATH-2 | 4 | 0 | 11 | 19 | 19 | 4 | 4 | 0 | 15 | 0 | 0 | 4 |
| SMAP-29 | 0 | 14 | 4 | 0 | 21 | 11 | 4 | 14 | 11 | 7 | 7 | 0 |
| mCRAMP | 0 | 15 | 6 | 6 | 3 | 24 | 0 | 9 | 12 | 3 | 0 | 0 |
| rCRAMP | 0 | 15 | 0 | 9 | 6 | 21 | 0 | 9 | 12 | 3 | 3 | 0 |
| PMAP-36 | 3 | 11 | 9 | 3 | 17 | 20 | 0 | 11 | 11 | 6 | 0 | 3 |
| LL-37 | 0 | 5 | 3 | 11 | 14 | 16 | 0 | 8 | 11 | 5 | 0 | 5 |
| CAP18-FV | 0 | 8 | 5 | 5 | 14 | 24 | 0 | 8 | 11 | 3 | 3 | 0 |
| PMAP-37 | 0 | 11 | 0 | 8 | 16 | 8 | 0 | 8 | 16 | 0 | 0 | 11 |
| ttLL-37 | 0 | 16 | 3 | 11 | 14 | 14 | 0 | 11 | 5 | 0 | 0 | 5 |
| eCATH-3 | 0 | 5 | 2 | 2 | 15 | 8 | 8 | 12 | 10 | 2 | 5 | 10 |
| Bac7 | 0 | 5 | **47** | 5 | **28** | 0 | 0 | 7 | 8 | 0 | 0 | 0 |
| Prophenin-1 | 1 | 9 | **53** | 19 | 8 | 0 | 0 | 1 | 0 | 1 | 1 | 0 |
| **Birds** | | | | | | | | | | | | |
| dCATH | 10 | 0 | 5 | 5 | 20 | 15 | 0 | 10 | 10 | 5 | 10 | 0 |
| CATHL3-CUCCA | 4 | 4 | 4 | 9 | 9 | 17 | 0 | 9 | 17 | 9 | 4 | 4 |
| chCATH-1 | 4 | 4 | 4 | 0 | 15 | 15 | 0 | 12 | 8 | 15 | 8 | 0 |
| chCATH-2 | 0 | 8 | 4 | 15 | **27** | 8 | 0 | 12 | 4 | 4 | 4 | 4 |
| Pc-CATH1 | 4 | 4 | 4 | 4 | 15 | 15 | 0 | 12 | 4 | 15 | 8 | 0 |
| CATHL3-AMAVI | 4 | 11 | 4 | 11 | 11 | 11 | 0 | 7 | 7 | 14 | 11 | 4 |
| chCATH-3 | 3 | 3 | 7 | 3 | 14 | 10 | 0 | 10 | 7 | 14 | 14 | 0 |
| cc-CATH3 | 3 | 3 | 7 | 3 | 17 | 7 | 0 | 10 | 7 | 14 | 14 | 0 |
| CATHL3-BUCRH | 3 | 0 | 7 | 7 | 7 | 17 | 3 | 7 | 7 | 14 | 7 | 3 |
| cc-CATH2 | 0 | 12 | 3 | 12 | 19 | 9 | 0 | 12 | 6 | 9 | 6 | 3 |
| CATHL2-COLVI | 0 | 16 | 3 | 12 | 22 | 6 | 0 | 6 | 9 | 9 | 3 | 0 |
| CATHL2-ACACH | 0 | 12 | 3 | 12 | 21 | 6 | 3 | 9 | 9 | 9 | 0 | 6 |
| Cl-CATH2 | 0 | 15 | 3 | 12 | **32** | 0 | 0 | 15 | 9 | 0 | 3 | 3 |
| CATHL2-PSEHU | 0 | 18 | 3 | 12 | 21 | 9 | 3 | 9 | 12 | 6 | 0 | 3 |
| CATHB1-CAPCA | 3 | 8 | 0 | 8 | 16 | 13 | 0 | 11 | 13 | 3 | 0 | 8 |
| chCATH-B1 | 10 | 5 | 10 | 2 | 18 | 2 | 2 | 10 | 8 | 5 | 0 | 5 |
| CATHB1-CHAVO | 2 | 7 | 5 | 12 | 12 | 14 | 0 | 10 | 10 | 0 | 2 | 2 |
| CATHB1-COLLI | 2 | 5 | 14 | 0 | 17 | 5 | 0 | 14 | 14 | 2 | 2 | 2 |
| **Reptiles** | | | | | | | | | | | | |
| As-CATH5 | 5 | 9 | 5 | 9 | 9 | 18 | 0 | 5 | 18 | 5 | 9 | 0 |
| As-CATH6 | 12 | 15 | 0 | 0 | 15 | 4 | 4 | 4 | 19 | 0 | 12 | 0 |
| Cathelicidin-BF | 0 | 3 | 7 | 17 | 10 | **30** | 0 | 7 | 3 | 10 | 3 | 7 |
| Hc-CATH | 0 | 3 | 3 | 10 | 17 | 23 | 0 | 3 | 20 | 7 | 3 | 7 |
| SA-CATH | 0 | 3 | 7 | 17 | 0 | **40** | 3 | 7 | 3 | 13 | 0 | 7 |
| CATHPb1 | 0 | 10 | 3 | 19 | 10 | **32** | 0 | 19 | 0 | 0 | 0 | 0 |
| As-CATH3 | 0 | 10 | 23 | 3 | 6 | 13 | 6 | 0 | 10 | 0 | 6 | 10 |
| Ps-CATH4 | 6 | 16 | 0 | 6 | **28** | 6 | 0 | 12 | 6 | 0 | 3 | 3 |
| Cm-CATH2 | 0 | 12 | 0 | 12 | **30** | 9 | 3 | 3 | 6 | 9 | 0 | 6 |
| Cm-CATH3 | 6 | 9 | 0 | 9 | 24 | 9 | 3 | 9 | 3 | 6 | 9 | 3 |
| NA-CATH | 0 | 3 | 6 | 21 | 6 | **38** | 0 | 3 | 3 | 9 | 3 | 3 |
| Ps-CATH6 | 0 | 0 | 17 | 8 | 0 | 17 | 0 | 0 | 3 | 8 | 11 | 8 |
| KP36 | 3 | 19 | 19 | 6 | 6 | 19 | 0 | 6 | 0 | 3 | 0 | 8 |
| As-CATH2 | 5 | 22 | 0 | 3 | **27** | 5 | 11 | 5 | 0 | 0 | 5 | 8 |
| As-CATH4 | 0 | 13 | 8 | 8 | 18 | 21 | 0 | 8 | 11 | 8 | 3 | 3 |
| Cm-CATH1 | 0 | 20 | 5 | 2 | 18 | 15 | 2 | 10 | 12 | 0 | 2 | 2 |
| Cm-CATH4 | 0 | 8 | 8 | 4 | 4 | 2 | 2 | 8 | 14 | 6 | 16 | 6 |
| As-CATH1 | 5 | 19 | 7 | 0 | 17 | 8 | 8 | 2 | 2 | 3 | 3 | 14 |
| **Amphibians** | | | | | | | | | | | | |
| Tylotoin | 0 | 0 | 0 | 0 | 17 | **25** | 0 | 0 | 0 | 17 | 0 | 0 |
| Cathelicidin-NV | 0 | 8 | 0 | 4 | 17 | 17 | 0 | 0 | 8 | 4 | 4 | 8 |
| Cathelicidin-OA1 | 4 | 4 | 7 | 4 | 4 | 7 | 7 | 7 | 11 | 0 | 7 | 7 |
| Cathelicidin-RC1 | 0 | 4 | 7 | 14 | 0 | **32** | 0 | 18 | 0 | 7 | 0 | 7 |
| Cathelicidin-PY | 0 | 3 | 3 | 3 | 10 | 17 | 3 | 7 | 14 | 7 | 0 | 3 |
| Lf-CATH1 | 0 | 10 | 10 | 7 | 13 | 3 | 0 | 10 | 3 | 7 | 7 | 13 |
| Lf-CATH2 | 0 | 17 | 3 | 0 | 7 | 10 | 3 | 7 | 13 | 10 | 0 | 13 |
| Cathelicidin-PP | 0 | 9 | 0 | 0 | 3 | 19 | 0 | 6 | 12 | 16 | 9 | 3 |
| Cathelicidin-RC2 | 0 | 15 | 0 | 9 | 6 | 18 | 3 | 3 | 6 | 3 | 6 | 9 |
| OL-CATH2 | 0 | 9 | 0 | 3 | 6 | 18 | 6 | 6 | 9 | 9 | 3 | 12 |
| OL-CATH1 | 0 | 9 | 9 | 6 | 12 | 9 | 0 | 9 | 3 | 6 | 0 | 12 |
| Cathelicidin-Bg | 6 | 9 | 12 | 0 | 18 | 6 | 0 | 3 | 6 | 3 | 6 | 6 |
| AdCath | 0 | 12 | 3 | 3 | 6 | 24 | 0 | 0 | 0 | 6 | 12 | 9 |
| BG-CATH37 | 3 | 14 | 11 | 0 | 22 | 5 | 0 | 3 | 5 | 5 | 0 | 8 |
| Cathelicidin-DM | 3 | 11 | 5 | 0 | 14 | 11 | 0 | 3 | 14 | 3 | 5 | 8 |
| Cathelicidin-AL | 0 | **48** | 0 | 0 | **25** | 0 | 2 | 2 | 0 | 2 | 6 | 12 |
| **Fish** | | | | | | | | | | | | |
| HFIAP-3 | 7 | 20 | 0 | 3 | 10 | 17 | 3 | 7 | 7 | 13 | 7 | 0 |
| HFIAP-1 | 5 | 11 | 0 | 5 | 16 | 14 | 5 | 0 | 5 | 11 | 8 | 0 |
| AdCATH | 0 | 21 | 5 | 3 | 18 | 8 | 0 | 0 | 8 | 5 | 0 | 16 |
| CATH-THYTH | 0 | **29** | 2 | 0 | 12 | 10 | 2 | 2 | 0 | 2 | 7 | 24 |
| JeCATH | 0 | **33** | 2 | 0 | 10 | 15 | 0 | 2 | 0 | 0 | 6 | 15 |
| CATH-ONCTS | 4 | **27** | 4 | 0 | 15 | 6 | 2 | 4 | 2 | 4 | 6 | 13 |
| AsCATH-2 | 2 | **30** | 6 | 2 | 15 | 8 | 0 | 2 | 0 | 0 | 6 | 19 |
| AcCATH | 0 | **28** | 4 | 2 | 17 | 4 | 2 | 2 | 0 | 2 | 8 | 19 |
| CATH-BRALE | 0 | **30** | 4 | 0 | 21 | 8 | 0 | 2 | 0 | 0 | 8 | 19 |
| CodCATH-2 | 0 | **27** | 4 | 0 | 24 | 7 | 0 | 2 | 5 | 2 | 0 | 20 |
| BtrCATH | 0 | **30** | 7 | 5 | 17 | 3 | 0 | 7 | 3 | 0 | 5 | 8 |
| aCATH | 0 | **38** | 2 | 0 | 7 | 16 | 0 | 2 | 0 | 0 | 3 | **26** |
| AsCATH-1 | 0 | 19 | 2 | 3 | 14 | 3 | 0 | 8 | 14 | 5 | 8 | 8 |
| rtCATH-2a | 2 | 21 | 9 | 2 | 9 | 8 | 3 | 3 | 3 | 3 | 5 | 14 |
| rtCATH-1 | 0 | **27** | 9 | 5 | 12 | 2 | 2 | 9 | 2 | 5 | 2 | 12 |
| CodCATH-1 | 0 | **35** | 1 | 0 | 20 | 4 | 0 | 1 | 0 | 0 | 3 | **25** |
| CodCATH-3 | 0 | **36** | 1 | 0 | 20 | 5 | 0 | 1 | 0 | 0 | 1 | **28** |
| CATH1-SALFO | 0 | **30** | 6 | 1 | 17 | 4 | 1 | 5 | 5 | 3 | 3 | 18 |

Trp = tryptophan; Gly = glycine; Pro = proline; Phe = phenylalanine; Arg = arginine; Lys = lysine; His = histidine; Ile = isoleucine; Leu = leucine; Val = valine; Ala = alanine; Ser = serine.

**Supplementary References**

1. Osorio D, Rondon-Villarreal P, Torres R. Peptides: A Package for Data Mining of Antimicrobial Peptides. *R J.* (2015) **7**:4–14.
2. R Core Team. *R: A Language and Environment for Statistical Computing*. Vienna, Austria: R Foundation for Statistical Computing (2020). Available at: <https://www.R-project.org/> [Accessed May 16, 2020]
3. Zanetti M. The role of cathelicidins in the innate host defenses of mammals. *Curr Issues Mol Biol* (2005) **7**:179–196.
4. Brahma B, Patra MC, Karri S, Chopra M, Mishra P, De BC, Kumar S, Mahanty S, Thakur K, Poluri KM, et al. Diversity, Antimicrobial Action and Structure-Activity Relationship of Buffalo Cathelicidins. *PLoS One* (2015) **10**:e0144741. doi:10.1371/journal.pone.0144741
5. Zhu S, Gao B. Positive selection in cathelicidin host defense peptides: adaptation to exogenous pathogens or endogenous receptors? *Heredity* (2017) **118**:453–465. doi:10.1038/hdy.2016.117
6. Cheng Y, Prickett MD, Gutowska W, Kuo R, Belov K, Burt DW. Evolution of the avian β-defensin and cathelicidin genes. *BMC Evol. Biol.* (2015) **15**:188. doi:10.1186/s12862-015-0465-3
7. Feng F, Chen C, Zhu W, He W, Guang H, Li Z, Wang D, Liu J, Chen M, Wang Y, et al. Gene cloning, expression and characterization of avian cathelicidin orthologs, Cc-CATHs, from *Coturnix coturnix*. *FEBS J.* (2011) **278**:1573–1584. doi:10.1111/j.1742-4658.2011.08080.x
8. Goitsuka R, Chen C-LH, Benyon L, Asano Y, Kitamura D, Cooper MD. Chicken cathelicidin-B1, an antimicrobial guardian at the mucosal M cell gateway. *Proc. Natl. Acad. Sci. U.S.A.* (2007) **104**:15063–15068. doi:10.1073/pnas.0707037104
9. Wang A, Zhang F, Guo Z, Chen Y, Zhang M, Yu H, Wang Y. Characterization of a Cathelicidin from the Colubrinae Snake, *Sinonatrix annularis*. *Zool. Sci.* (2019) **36**:68–76. doi:10.2108/zs180064
10. Chen Y, Cai S, Qiao X, Wu M, Guo Z, Wang R, Kuang Y-Q, Yu H, Wang Y. As-CATH1-6, novel cathelicidins with potent antimicrobial and immunomodulatory properties from *Alligator sinensis*, play pivotal roles in host antimicrobial immune responses. *Biochem. J.* (2017) **474**:2861–2885. doi:10.1042/BCJ20170334
11. Qiao X, Yang H, Gao J, Zhang F, Chu P, Yang Y, Zhang M, Wang Y, Yu H. Diversity, immunoregulatory action and structure-activity relationship of green sea turtle cathelicidins. *Dev. Comp. Immunol.* (2019) **98**:189–204. doi:10.1016/j.dci.2019.05.005
12. Tankrathok A, Punpad A, Kongchaiyapoom M, Sosiangdi S, Jangpromma N, Daduang S, Klaynongsruang S. Identification of the first *Crocodylus siamensis* cathelicidin gene and RN15 peptide derived from cathelin domain exhibiting antibacterial activity. *Biotechnol. Appl. Biochem.* (2019) **66**:142–152. doi:10.1002/bab.1709
13. Mu L, Tang J, Liu H, Shen C, Rong M, Zhang Z, Lai R. A potential wound-healing-promoting peptide from salamander skin. *FASEB J.* (2014) **28**:3919–29. doi:10.1096/fj.13-248476
14. Wu J, Yang J, Wang X, Wei L, Mi K, Shen Y, Liu T, Yang H, Mu L. A frog cathelicidin peptide effectively promotes cutaneous wound healing in mice. *Biochem. J.* (2018) **475**:2785–2799. doi:10.1042/BCJ20180286
15. Cao X, Wang Y, Wu C, Li X, Fu Z, Yang M, Bian W, Wang S, Song Y, Tang J, et al. Cathelicidin-OA1, a novel antioxidant peptide identified from an amphibian, accelerates skin wound healing. *Sci. Rep.* (2018) **8**:943. doi:10.1038/s41598-018-19486-9
16. Yu H, Cai S, Gao J, Zhang S, Lu Y, Qiao X, Yang H, Wang Y. Identification and polymorphism discovery of the cathelicidins, Lf-CATHs in ranid amphibian (*Limnonectes fragilis*). *FEBS J.* (2013) **280**:6022–32. doi:10.1111/febs.12521
17. Ling G, Gao J, Zhang S, Xie Z, Wei L, Yu H, Wang Y. Cathelicidins from the bullfrog *Rana catesbeiana* provides novel template for peptide antibiotic design. *PloS One* (2014) **9**:e93216. doi:10.1371/journal.pone.0093216
18. Qi R-H, Chen Y, Guo Z-L, Zhang F, Fang Z, Huang K, Yu H-N, Wang Y-P. Identification and characterization of two novel cathelicidins from the frog *Odorrana livida*. *Zool Res* (2019) **40**:94–101. doi:10.24272/j.issn.2095-8137.2018.062
19. Gao F, Xu WF, Tang LP, Wang MM, Wang XJ, Qian YC. Characteristics of cathelicidin-Bg, a novel gene expressed in the ear-side gland of *Bufo gargarizans*. *Genet. Mol. Res.* (2016) **15**: doi:10.4238/gmr.15038481
20. Yang H, Lu B, Zhou D, Zhao L, Song W, Wang L. Identification of the first cathelicidin gene from skin of Chinese giant salamanders *Andrias davidianus* with its potent antimicrobial activity. *Dev. Comp. Immunol.* (2017) **77**:141–149. doi:10.1016/j.dci.2017.08.002
21. Sun T, Zhan B, Gao Y. A novel cathelicidin from *Bufo bufo gargarizans* Cantor showed specific activity to its habitat bacteria. *Gene* (2015) **571**:172–7. doi:10.1016/j.gene.2015.06.034
22. Shi Y, Li C, Wang M, Chen Z, Luo Y, Xia X-S, Song Y, Sun Y, Zhang A-M. Cathelicidin-DM is an Antimicrobial Peptide from *Duttaphrynus melanostictus* and Has Wound-Healing Therapeutic Potential. *ACS Omega* (2020) **5**:9301–9310. doi:10.1021/acsomega.0c00189
23. Chen Y, Gong Q, Song M, Lai J, Sun J, Liu Y. Identification and characterization of three novel antimicrobial peptides from *Acipenser dabryanus*. *Fish Shellfish Immunol.* (2019) **88**:207–216. doi:10.1016/j.fsi.2019.02.050
24. Scocchi M, Pallavicini A, Salgaro R, Bociek K, Gennaro R. The salmonid cathelicidins: a gene family with highly varied C-terminal antimicrobial domains. *COMP BIOCHEM PHYS B* (2009) **152**:376–81. doi:10.1016/j.cbpb.2009.01.003
25. Masso-Silva JA, Diamond G. Antimicrobial peptides from fish. *Pharmaceuticals* (2014) **7**:265–310. doi:10.3390/ph7030265
26. Zhang X-J, Zhang X-Y, Zhang N, Guo X, Peng K-S, Wu H, Lu L-F, Wu N, Chen D-D, Li S, et al. Distinctive structural hallmarks and biological activities of the multiple cathelicidin antimicrobial peptides in a primitive teleost fish. *J. Immunol.* (2015) **194**:4974–4987. doi:10.4049/jimmunol.1500182
27. Chang C-I, Pleguezuelos O, Zhang Y-A, Zou J, Secombes CJ. Identification of a novel cathelicidin gene in the rainbow trout, *Oncorhynchus mykiss*. *Infect. Immun.* (2005) **73**:5053–5064. doi:10.1128/IAI.73.8.5053-5064.2005
28. Wang G, Li X, Wang Z. APD3: the antimicrobial peptide database as a tool for research and education. *Nucleic Acids Res.* (2016) **44**:D1087–93. doi:10.1093/nar/gkv1278
